# Application of a locally developed open-access digital monitoring system for the human milk bank network in Vietnam

**DOI:** 10.1186/s13006-025-00745-1

**Published:** 2025-07-08

**Authors:** Tuan Thanh Nguyen, Hoang Thi Tran, Khoa Thanh Nhat Tran, Oanh Thi Xuan Nguyen, Anh Tu Thi Nguyen, Roger Mathisen

**Affiliations:** 1 Alive & Thrive, FHI 360 Global Nutrition, Hanoi, Vietnam; 2 College of Health Sciences, VinUniversity, Hanoi, Vietnam; 3 Da Nang Hospital for Women and Children, Da Nang, Vietnam; 4 University of Da Nang, Da Nang, Vietnam; 5 Phuc Hai Dang Co. Ltd., Da Nang, Vietnam; 6 Tu Du Hospital, Ho Chi Minh City, Vietnam

**Keywords:** Breastmilk, Donor milk, Health system strengthening, Human milk bank, Preterm infant feeding, Localization, Low birthweight, Monitoring system, Preterm births

## Abstract

**Background:**

Establishing the first Human Milk Bank (HMB) in Vietnam at the Da Nang Hospital for Women and Children marks a significant advancement in neonatal care. This specialized service addresses the critical need for donor human milk (DHM) when a mother's own milk is unavailable, providing essential nutrition to small vulnerable newborns. Introducing the new specialized service required developing a digital monitoring system to ensure the quality and safety of DHM.

**Development of the tool:**

This digital and open access system was designed using an approach called DMADV (Define, Measure, Analyze, Design, and Verify), and includes features for human milk donor management, DHM processing, recipient management, and real-time data access. It has streamlined operations, enhanced efficiency, and ensured adherence to safety and quality standards.

**The use of the tool:**

The application of the monitoring system has facilitated the tracking of DHM from collection to use, ensuring transparency and accountability. Data collected through this system has been instrumental in improving program performance, informing decisions, and supporting research. The successful scaling up of HMB services and the monitoring system to other regions in Vietnam demonstrates the project's effectiveness and potential for broader impact. The system's adaptability has allowed continuous improvements and integration of new functions, such as financial reporting and consumables tracking.

**Lessons learned:**

Lessons learned from the Da Nang HMB have been shared to guide the development of HMB monitoring systems in other regions and countries. The digital monitoring system has proven to be a critical tool in maintaining the quality and safety of DHM, ultimately contributing to better health outcomes for children. The successful implementation of HMB services underscores the importance of robust monitoring systems in its operations and highlights the potential for digital solutions to enhance healthcare services.

**Supplementary Information:**

The online version contains supplementary material available at 10.1186/s13006-025-00745-1.

## Background

Breastfeeding and human milk are the normative standards for infant and young child feeding [1]. When a mother’s own milk is not available, donor human milk (DHM) from a human milk bank (HMB) is the recommended alternative by the World Health Organization (WHO) for low birthweight infants [2]. HMBs recruit donors; collect, store, screen, and pasteurize raw breastmilk; and administer DHM to newborns in need [3, 4]. HMB services play a crucial role in neonatal care, providing essential nutrition to infants who are unable to receive breastmilk from their mothers [3, 4]. These services offer life-saving protection, particularly for premature and low-birth-weight infants [3, 5]. By ensuring that small vulnerable infants receive the necessary nutrition for their survival, growth, and development, HMB services help reduce the risk of infections and other health complications [5].

HMB services are based on five core components: 1) safety, 2) quality, 3) networking and information sharing, 4) awareness, advocacy, promotion, and 5) sustainability [4]. Safety focuses on reducing contaminants, conducting regular audits, and ensuring policy compliance. Quality highlights maintaining the biological and nutritional integrity of DHM through operational and managerial consistency. Networking and information sharing promote activity transparency, while awareness and advocacy support lactation and breastfeeding counseling and policy alignment. Sustainability ensures a balanced supply and demand, financial integrity, and stakeholder engagement [4]. Foundational activities like quality assurance, auditing, and guidance for clinical provisions support these pillars [4].

A robust monitoring system is critical for the successful operation of HMBs [6, 7]. Such a system ensures that all foundational activities and key pillars of an HMB, including donor recruitment, milk collection, processing, storage, and distribution, are carried out efficiently and safely [7]. Monitoring systems help maintain high standards of quality and safety, ensuring that the DHM is free from contaminants and meets all nutritional requirements [7]. Additionally, these systems facilitate the tracking of DHM from collection to distribution, ensuring transparency and accountability [7].

Despite the recognized importance of monitoring systems in HMBs, there is limited information available about their status and how data are utilized globally [6]. Our PubMed search using the keywords (((breast) OR (human)) AND (milk bank)) AND (monitor) resulted in only 83 papers as of December 25, 2024. However, none of these papers describe their monitoring systems, except for some studies that mention the sources of data used for their analyses. This gap in knowledge hinders the ability to optimize HMB operations and improve outcomes for small vulnerable infants. Understanding the current state of monitoring systems and their data usage is essential for identifying best practices and areas needing improvement, which can ultimately enhance the effectiveness of HMBs worldwide [6].

In this manuscript, we report our experience in establishing, operating, and scaling up the locally developed, open access digital monitoring system for the first HMB in Vietnam. Our journey provides valuable insights into the challenges and successes encountered during implementation. By sharing our experience, we aim to contribute to the global knowledge base on HMB monitoring systems and offer practical guidance for other HMBs seeking to enhance their operations.

## Introduction about the HMB in Da Nang

### Establishment

The establishment of the first human milk bank in Vietnam has been described in detail elsewhere [7]. It was established within the Da Nang Hospital for Women and Children, designated as a Center of Excellence for Early Essential Newborn Care (EENC) and Kangaroo Mother Care (KMC) in Vietnam. This hospital is the highest-tier national referral hospital responsible for district hospitals in Da Nang and other South and Central provinces. This hospital also champions breastfeeding promotion, protection, and support, including launching provincial franchise services to provide counseling on maternal, infant, and young child nutrition and the Ministry of Health designation as a Center of Excellence for Breastfeeding. Each year, there are 13,000–15,000 births at this hospital. The hospital’s neonatal unit, the highest-tier level (level 4), provides treatment for between 4,000–5,000 infants each year [7].

### Operation

**Fig. 1:**
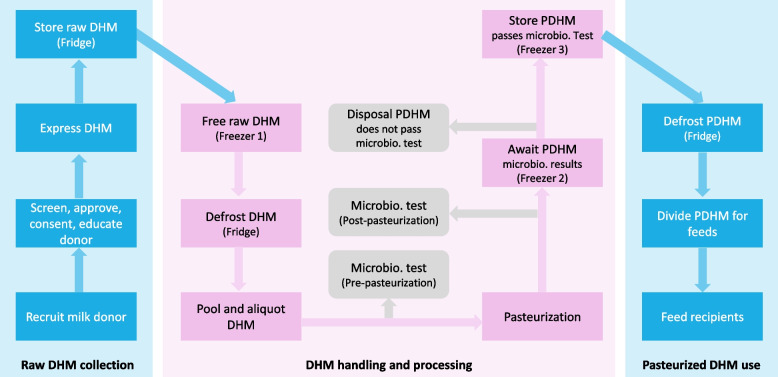
Operation diagram of the human milk bank in Da Nang. Abbreviations: Microbio.: Microbiological, DHM: Donor human milk; PDHM: Pasteurized DHM

Da Nang HMB applies high standards to serve as a pilot and knowledge leadership and learning model in Vietnam. Every step in the HMB process (Fig. 1) is detailed in operation manuals and Standard Operating Procedures (SOPs). The manuals and SOPs cover various components of HMB practices, including donor recruitment, donor screening, hygiene practice, breastmilk expression and handling, storage, pasteurization, microbial testing, and the distribution through a cold chain of DHM to the neonatal unit and postnatal wards [7]. There were also several monitoring forms, assuming this was a fully paper-based HMB monitoring system (Additional file 1).

## Development of HMB monitoring system

**Fig. 2:**
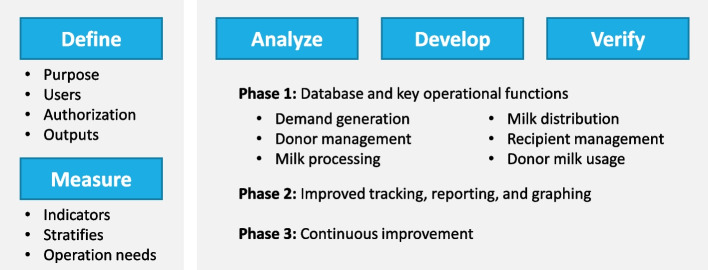
Development procedure based on the Define, Measure, Analyze, Design, and Verify (DMADV) model

We used an approach known as DMADV (Define, Measure, Analyze, Design, and Verify) for the development of the digital HMB monitoring system [8] (Fig. 2).

### Define

#### The monitoring system purpose

We aimed to create a user-friendly, digitized HMB monitoring system to align with the guidelines and SOPs and to provide real-time data to optimize the operation of the Da Nang HMB. Such a system ensures that all activities within the HMB follow standardized protocols, which is crucial for maintaining the quality and safety of the donor milk. The HMB can ensure transparency and accountability by implementing a system that tracks and traces DHM from donation to use. This enhances the efficiency of HMB’s operations and builds trust among donors and recipients.

#### Users

The system is designed to be used by health workers from different units in the hospital, primarily nurses and midwives, who are integral to the daily operations of the HMB. Given the varying levels of IT competency among these users, the system must be intuitive and easy to navigate, ensuring that even those with low to moderate IT skills can use it effectively. This inclusivity is vital for the smooth functioning of the HMB, as it allows all staff members to contribute to and benefit from the system’s capabilities.

#### Authorization

Authorization within the system using usernames and passwords is structured to ensure that only appropriate personnel have access to specific functions based on their roles. Managers, HMB staff, and staff from postnatal wards and the neonatal units are granted access according to their functions. This role-based access control helps maintain data security and integrity, ensuring that sensitive information is only accessible to those who need it for their work.

#### Tool outputs

The system operates online, providing real-time data access and updates. The key functions required by the HMB, such as donor milk tracking, protocol adherence, and data reporting, are all supported by this monitoring system.

### Measurement

The indicators have been carefully mapped to align with the operational procedures of the HMB (Fig. 1) [7]. These indicators are categorized into key groups to ensure comprehensive monitoring and evaluation of the HMB’s activities (Table 1). The first group focuses on recruiting, screening, and managing donors and their DHM, which is crucial for maintaining a reliable supply of safe DHM. The second group encompasses receiving, storage, processing, and distribution of DHM within the HMB. These processes are essential for ensuring that DHM retains its nutritional and immunological properties while being safe for consumption. Both groups of indicators are further stratified based on the source of DHM, whether it is obtained from the hospital or the community, to provide a detailed understanding of the DHM’s journey from donor to recipient.

**Table 1:** Indicators of the operational report

From date….. month………year 20…. To date….. month………year 20….				
1. Demand generation and donor recruitment	Total	In the hospital	Community	Other
1.1. Number of group counseling sessions for demand generation (≤ 10 mothers)				
1.2. Number of demand generation events (> 10 mothers)				
1.3. Number of lactating mothers who attended donor recruitment sessions (total):				
1.3.1. One-on-one sessions				
1.3.2. Group sessions				
1.3.3. Events				
1.4. Number of mothers who expressed interest in donating after recruitment				
1.5. Number of mothers newly screened				
1.6. Number of lactating mothers newly screened and meeting all eligibility requirements to become human milk donors				
1.7. Number of eligible human milk donors trained in proper hygiene and donation skills (e.g., handwashing, safe milk expression, storage, labeling, and transport to the HMB)				
1.8. Number of new donors who donated milk				
1.9. Number of mothers who stopped donating milk (e.g., due to child age above 9 months, disease status, personal decision, or absence of milk donation for 90 days at this HMB)				
2. Receiving, storage, processing, and distribution of donor milk				
2.1. Volume of milk donated (L)				
2.2. Volume of pasteurized donor human milk (DHM) (L):				
2.2.1. Passed pre-pasteurization test (L)				
2.2.2. Passed post-pasteurization test (L)				
2.2.3. Passed both pre- and post-pasteurization tests (L)				
2.3. Volume of donor milk disposed of for any reason (L)				
2.4. Total volume of pasteurized DHM available at the HMB at the reporting time (L)				
3. Usage of pasteurized DHM		Neonatal units	Postnatal wards	Other
3.1. Volume of distributed pasteurized DHM (L)				
3.2. Number of children who started receiving pasteurized DHM from the HMB				
3.3. Number of children currently receiving milk from the HMB				
3.4. Volume of pasteurized DHM fed to infants (L)				
3.5. Average number of days pasteurized DHM was used (for infants who stopped receiving DHM during the reporting period)				
3.6. Amount of money received (thousand VND)				

The usage of pasteurized DHM is monitored across different hospital units, including neonatal units, postnatal wards, and post-operative wards (Table 1). This stratification allows for a detailed analysis of how DHM is utilized in various clinical settings, ensuring that the DHM is used efficiently and effectively to meet the needs of different patient populations. By tracking these indicators, the HMB can identify areas for improvement, optimize its operations, and ultimately enhance the quality of care provided to infants who rely on DHM for their survival, nutrition, and growth.

### Analyze development options and develop, verify, and refine

#### Phase critical components

The HMB monitoring system is designed with three critical phases to ensure its functionality from day one (Fig. 2). The first phase involves collecting and managing information, which is crucial for building a reliable database. The second phase focuses on reporting and graphing, providing visual insights and comprehensive reports with key indicators for better decision-making. The third phase involves enhancing and adding new components to the system, ensuring continuous improvement and adaptability to meet evolving needs.

This monitoring system allows for the export of monitoring data for further in-depth analyses using statistical packages. Therefore, we did not plan to build analyses other than stratified descriptive analysis into this system. This will help reduce the required development, testing, and maintenance efforts and make the system lighter to fit the local infrastructure.

#### Testing forms and procedures

The informed consent form and agreement for use have been rigorously tested with mothers of newborns in neonatal units and postnatal wards. These finalized forms have been integrated into the digital monitoring tool, ensuring that all necessary documentation is accurately captured and easily accessible. This testing phase has been crucial in refining the forms and procedures to meet the needs of both healthcare providers and patients.

#### Digital and complementary paper-based system

While the digital system aims to minimize paper-based forms, certain documents still require physical copies due to the need for signatures or auditing purposes. These include informed consent forms, pasteurization procedures, testing results, and approvals for pasteurized DHM use. Recognizing the shift towards digital data, we developed comprehensive forms within the online monitoring system, allowing users to print completed copies for signatures. Over time, the number of required paper-based forms has been significantly reduced, streamlining operations and improving efficiency. For more details on this transition, please refer to the later sections of this document.

#### Testing on the label/QR code for donor and container to maximize efficiency

To enhance efficiency, we have developed QR codes for tracking and tracing. Testing labels and QR codes for donors and containers include several key components to optimize cost savings. Firstly, a single label for raw DHM for each donor is printed for the storage box in the freezer, utilizing regular paper. Additionally, the design process encompasses creating a donor ID card and corresponding QR code and developing labels and QR codes for the containers using a more expensive, heat- and water-resistant paper. Given the expensive paper, we minimize the information stored in the QR codes and the information on the labels to reduce the area required for the label. We also created an option to print multiple labels to reduce white, unnecessary space.

#### Information technology (IT) platform and software developer

This web-based monitoring system was developed using Microsoft technologies, built on an ASP.NET framework and hosted on a Windows Server environment with IIS, and utilizes Microsoft SQL Server 2008 R2 for the backend database. A more detailed visualization of the general database structure, including database relation and function flow versus database, is presented in Additional file 2, which translated all paper-based forms into Additional file 1 to digital forms. The minimal technical infrastructure required includes a Windows-based SQL Server 2008 R2 or later, client devices with browsers, stable internet connectivity, and an uninterrupted power supply for the server from the IT company (between 2017–2024) and hospital server (from 2025).

Microsoft SQL Server 2008 R2 is a robust and feature-rich database management system that supports various data-driven applications. Key features include support for relational, XML, and spatial data and advanced reporting and analytics through SQL Server Reporting Services. The system also offers enhanced security features and better management tools, making it a reliable choice for both small and large-scale enterprise applications. The software developer is Phuc Hai Dang Co. Ltd (PHD Ltd). Established in 2011, PHD Ltd specializes in building information systems across various platforms, including websites, personal computers, and mobile devices. The company has completed numerous relevant projects, such as e-hospital, e-clinic, e-office, and e-training systems in central provinces like Da Nang, Quang Nam, and Quang Tri. Notably, PHD Ltd has collaborated with Da Nang Hospital for Women and Children on several projects, including the ongoing Neonatal Screening Project, which links the screening unit with other hospital units and clinics. Their close working relationship with the hospital provides advantages such as familiarity with its system and potential cost reductions by utilizing existing infrastructure. Based in Da Nang, PHD Ltd can also offer timely and cost-effective training and support to HMB staff.

#### Testing, verifying, and refining

The testing process corresponds to various phases, including the initial (collecting information) and improved versions. The development team (PHD Ltd, Alive & Thrive, the hospital, and PATH) tests tool operation, reports, and graphs for any changes. The team enters and uses numbers in various scenarios to test the tool, ensuring the accuracy of values along the chain from donation to usage, as well as in reports and graphs. After testing, HMB and hospital staff deploy the monitoring tool or its updates. We continue gathering user feedback to improve the tool, adjusting based on evolving needs.

Regarding feedback mechanisms, in the initial stage, staff from PHD Ltd, Alive & Thrive, the hospital, and PATH communicated face-to-face to improve the HMB monitoring tool. This included direct observation, interviews, discussions about feasible solutions, and implementing the changes. Later, we created a social media group (using Zalo) for users to report issues or suggest improvements, allowing PHD Ltd or other parties to address them promptly. After approximately one year of operation, the HMB staff work directly with the IT company to manage, sustain, and improve the monitoring system. The hospital pays a fee per their agreement with the IT company.

Da Nang Hospital for Women and Children has been piloting a digital medical record system. The HMB monitoring system has recently been reallocated to the hospital server with plans for integration into digital medical records.

### Cost for the establisment and maintainnace of the HMB monitoring system in Da Nang

According to our records, the contract value with PHD Ltd was about USD 2,500, with an additional USD 1,000 for major improvements over the subsequent years. Hosting and continuous support were kindly contributed by PHD Ltd but would be approximately an annual fee of USD 100 (or a monthly fee of USD 8) based on the full handover of the HMB monitoring system to the hospital. Based on denominators from the costing study for the establishment and operation of Da Nang HMB [9], this startup expenditure is about 1% of the lowest estimation of costs for establishing Da Nang HMB (Scenario 3: Total processing cost (capital costs + implementation costs)) and 0.1% of the monthly costs of about USD 9,000.

## Key features and contributions to strengthen HMB operations

**Fig. 3:**
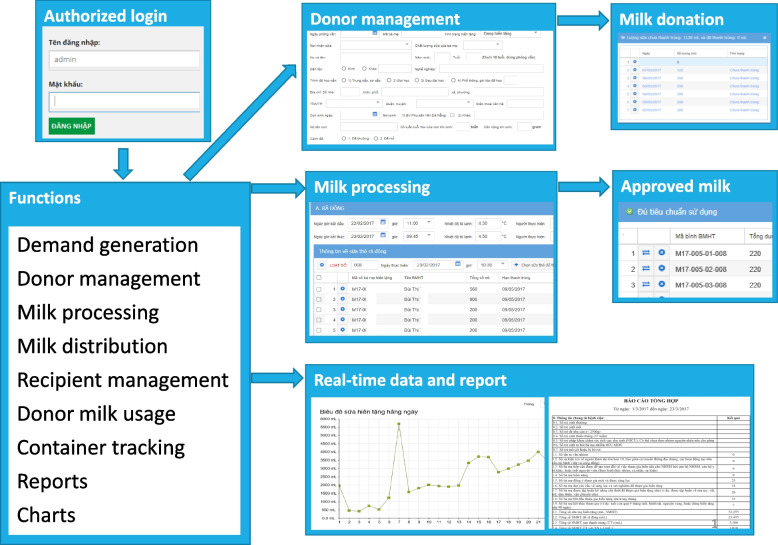
Key components of the human milk bank monitoring system

The HMB monitoring system is a web-based system that allows access from computers, tablets, and smart phones. The systems have been tested and functioning in popular web browsers in Vietnam, such as MS Internet Explorer/Edge, Chrome, and Safari in Windows and IOS operating systems. Key features were visualized in Fig. 3 and Table 2.

**Table 2:** Examples of the functions and potential usages of the HMB monitoring system and authorized users

		Authorized users		
The functions	The usages	HMB Manager	HMB staff	Neonatal or postnatal units
1. Raw DHM collection				
Demand generation management	Record the number and types of demand generation activities and their outputs		√	√
HMB donor management	Record the characteristics of donors Track donors through all stages: recruitment, screening (behavioral questions and serological tests), approval, consent, education, and donation (start and end dates)		√	
Donation tracking	Track each donor’s donations over time, including volume, date of expression, time, and storage conditions prior to delivery to the HMB		√	
2. DHM handling and processing				
Tracks the amount and expiration date of raw milk from each donor	Decide when to pasteurize milk from each donor		√	
Estimates the number of containers needed and prints container IDs	Assign raw milk to containers and print container IDs		√	
Monitors donor milk processing	Record and monitor milk processing conditions, including defrosting, pooling, and pasteurization		√	
Manages microbiological tests	Enter laboratory results and decide whether the milk should be used or disposed of		√	
Pasteurized DHM approval	Give decision on pasteurized DHM approval	√		
Tracks the amount and expiration date of pasteurized DHM from each donor	Determine when to use pasteurized DHM from each donor		√	
Track the amount of raw DHM at the HMB	Decide on the optimal timing for recruiting additional donors		√	
Track the amount of pasteurized DHM in the HMB	Decide when to pasteurize raw DHM		√	
3. Pasteurized DHM use				
Recipient management	Record the characteristics of each recipient		√	√
Ordering and order processing	Assist health workers in postnatal, neonatal, and post-operative units in requesting pasteurized DHM for each recipient Approve the orders submitted by HMB staff		√	√
Split pasteurized DHM for use by each child	Provide reminders for the number of feeds and the volume of pasteurized DHM required for each recipient. Print usage labels for each child		√	
Track the use of pasteurized DHM from each recipient	Track each recipient’s usage over time, including volume used, and the date and time of usage		√	√
4. Other functions				
Notifications and alerts	Display pop-up messages to flag potential data entry errors or inconsistent date ranges Use color-coded alerts to highlight critical information (e.g., the DHM supply approaching 8 L, raw DHM nearing its pasteurization deadline, or pasteurized DHM nearing its expiration)	√	√	√
Reports and analysis	Provide summaries of key indicators for any specified period Allow data to be downloaded for further analysis and reporting	√	√	
Tracking and Tracing through ID and QR codes of the mothers’ cards and labels of containers	Enable tracking and tracing across the entire process: donors, raw DHM, pasteurization, pasteurized DHM, and recipients		√	
User management	User roles and permissions User registration and authentication User activity monitoring Set/modify user notifications and alerts	√	√	

The users were assigned functions and units to allow them to access what was relevant to them (Table 2). For example, neonatal departments include units A, B, and C; the first unit will have the username set to Unit A. This username will only show information, functions, and user rights related to Unit A. HMB staff may take different roles, giving them access to most of the functions. The key role of the HMB manager is to make decisions on pasteurized DHM approval, assign roles to select staff/units, and review reports to oversee HMB operations and make decisions (Table 2). The app developer can modify and improve the system (not the data) upon request by the HMB manager. The external IT firm needs permission from the hospital’s IT manager and works under supervision when modifying the HMB tool hosted on the hospital server.

The HMB software efficiently manages donor recruitment by recording the number and type of activities and outputs. It also handles donor management by recording donor characteristics and tracking them through all processes, including recruitment, screening with behavioral questions and serological tests, approval, consent, education, and donation with start and end dates. Additionally, it tracks each donor’s donations over time, noting the volume, date expressed, date, time, and storage conditions before sending them to the HMB (Fig. 3).

Regarding DHM handling and processing, the software tracks the amount and expiration date of raw DHM from each donor, deciding when to pasteurize it. It estimates the number of containers needed, assigns raw DHM to containers, and prints container IDs. The system records and monitors DHM processing conditions, including defrosting, pooling, and pasteurization, while managing microbiological tests by entering lab results and deciding whether the DHM should be used or disposed of. It also tracks the amount and expiration date of pasteurized DHM from each donor, determining when to use it. Additionally, the software tracks the amount of raw and pasteurized DHM at the HMB, deciding on the timing of recruiting additional donors and pasteurizing raw DHM (Fig. 3).

The software effectively manages recipient information by recording the characteristics of each recipient and assisting health workers in postnatal, neonatal, and post-operation units by requesting pasteurized DHM for each recipient. It approves orders from HMB staff and helps split pasteurized DHM for use by each child, reminding the number of feeds and the volume of pasteurized DHM needed. Additionally, it prints labels for each child regarding the use of pasteurized DHM and tracks each recipient’s usage over time, including the volume used, date, and time of use (Fig. 3).

The system provides comprehensive automated notifications and alerts for all the above functions to ensure data accuracy and timely actions. It includes pop-up messages for potential typos in data ranges and color-coded information highlighting critical points needing attention. For example, it alerts users when the amount of DHM is approaching 8 L, when it’s time for raw DHM to be pasteurized, and when pasteurized DHM needs to be used. There is also a notification when the raw or pasteurized DHM falls below a certain threshold, triggering the need to recruit more mothers or perform pasteurization of the raw DHM.

The software’s reporting and analysis function provides comprehensive summaries of key indicators for any specified period, enabling users to monitor and evaluate performance effectively (Fig. 2, Table 1). It also allows for data downloading, facilitating further analysis and use in various applications.

**Fig. 4:**
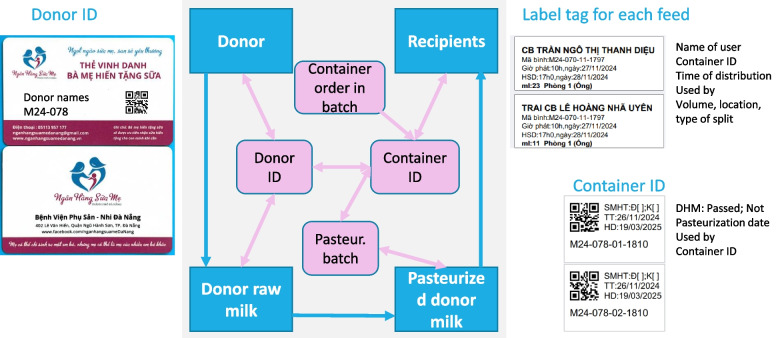
Tracking and tracing of donor human milk (DHM)

The software enables comprehensive tracking and tracing (Fig. 4) through ID and QR codes on mothers’ cards and container labels, ensuring seamless monitoring among donors, raw DHM, pasteurization processes, pasteurized DHM, and recipients. For instance, if a recipient becomes sick, the system can identify the donor milk containers received, the pasteurization batch and conditions, the donor(s), and other recipients from the same container. If a donor’s health condition changes, the system can trace her DHM and the recipients. Additionally, if a milk sample fails a lab test, the system can pinpoint potential reasons, including donor, staff, and equipment issues.

The mothers’ card (Fig. 4) serves multiple purposes. Honor the donor—we named it the “Card to Honor the Mothers Who Donate Breastmilk.” It includes the name, ID number, and a QR code. It highlights the key benefit of prioritizing receiving DHM when needed in the future. It is also an effective measure to promote the HMB by featuring its logo, slogans, address, and contact information. This credit card-sized card can be easily kept in a wallet.

Instructions and user manuals have also been developed (Additional file 3, only available in Vietnamese). The main purpose of these instructions is to guide the use of the HMB monitoring tool. They also help hospitals understand what needs to be done with their monitoring when they wish to establish an HMB.

The design of the system has been reviewed and approved by hospitals according to Vietnam regulations and FHI 360 Data Privacy and Protection Policy, which is aligned with the EU’s General Data Protection Regulation (GDPR). The operation of the HMB monitoring system strictly follows national and international data protection regulations. Additionally, each time we conduct research regarding data from the HMB, we obtained appropriate local IRB approval. Informed consents have already been integrated into the standardized consents for donors, covering the use of demographic information and donation details of donors, as well as the representative of users regarding their child’s demographic and clinical information and the use of donor human milk for statistics and research. This helps improve the human milk bank’s operations and create knowledge (Additional file 1).

## Evidence of data usage for decision-making

The Da Nang HMB was established in February 2017 with financial and technical support from the Vietnam Ministry of Health, Da Nang Department of Health, Da Nang Hospital for Women and Children, Alive & Thrive, PATH, and international experts [7]. The Da Nang HMB has been operational since its inception, even during the COVID-19 pandemic.

**Fig. 5:**
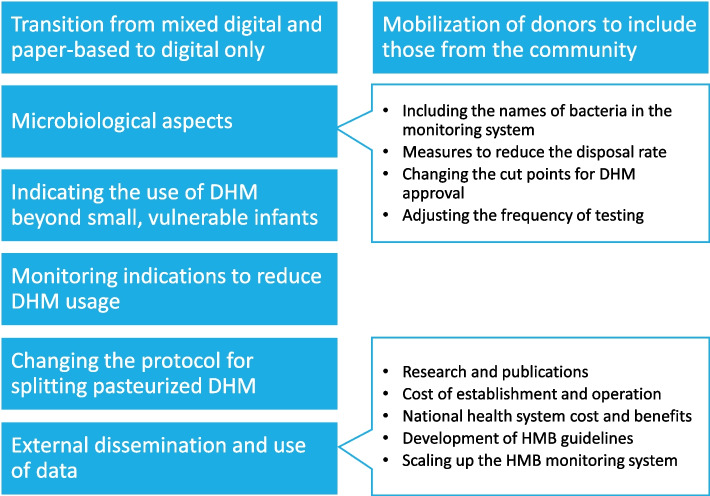
Evidence of data usage for decision-making

The HMB monitoring system was designed in 2016 and has been in use since the HMB's establishment, continuing to be improved throughout its operation. Since the planning stage of establishing the HMB, data have been systematically collected and used to improve program performance, inform decisions, and share knowledge (Fig. 5).

### Transitioning from mixed digital and paper-based to digital-only

Using paper-based forms was deemed necessary, especially during the early data collection and processing stages. The software systems were designed to accommodate both digital and paper-based forms. However, as the system matured, there was a gradual shift towards exclusively electronic forms. This transition not only streamlined the data collection process but also enhanced the efficiency and accuracy of data management.

### Mobilization of donors to include those from the community

Efforts to mobilize donors expanded from mothers in neonatal units to hospital staff and the broader community. Initial concerns included the ability to obtain high-quality DHM from outside the hospital. Some hospital staff with infants were also willing to donate their breastmilk. We extended the program to them because they have good knowledge and skills and operate within the controlled environment of the hospital. There was also a demand for donors within the hospital willing to provide DHM. We developed SOPs to monitor the quality of DHM and collect it from the community. During the process, we collected and monitored the quantity and bacterial profile of DHM. The results showed that these expansions resulted in a higher quantity of DHM and a lower disposal rate, making the process more efficient. The surplus pasteurized DHM could support other hospitals within Da Nang and HMB service in Quang Nam and Quang Tri provinces, ensuring a wider reach and better resource utilization.

### Microbiological aspects

#### Including the names of bacteria in the monitoring system

The initial process involved including only the bacteria count (i.e., number of CFU/mL). However, at a later stage, there is a need to identify the type of bacteria, as certain bacteria should not be present while others can be present in specific quantities. Options for bacteria names were included, allowing HMB staff to click on the names of frequently found bacteria in raw and pasteurized DHM. This inclusion helps the HMB manager and staff make more informed decisions about DHM approval and corrective actions without adding more work for the HMB staff.

#### Measures to reduce the disposal rate

High disposal rates were initially a concern, often due to water quality and procedures at the microbiological unit. Mapping the microbiological profile helped identify the causes and suggest necessary actions regarding donors, the donation process, storage of raw DHM, processing, and microbiological test procedures. These measures decreased the disposal rate from almost 40% in the first few months of operation to less than 10% after the first year.

#### Changing the cut points for DHM approval

The criteria for approving pasteurized DHM were revised, changing from a strict 0 CFU/mL to a more lenient < 10 CFU/mL. This adjustment was based on regular monitoring of DHM with post-pasteurization tests showing 1–9 CFU/mL, first used for older children and then for newborns other than preterm and sick newborns. This helped reduce unnecessary disposal while maintaining safety standards.

#### Adjusting the frequency of testing

Consistence in testing results allowed certain tests to be skipped, streamlining the process without compromising safety. This change helped to optimize resource use and reduce operational burdens.

### Indicating the use of DHM beyond small vulnerable newborns

Surplus DHM was also utilized for healthy newborns in postnatal units, providing essential nutrition when mothers’ own milk was not yet present. This was done while intensive lactation counseling and support were also provided to demonstrate the transition from DHM to breastfeeding. Giving DHM to those newborns is an effective way to reduce the use and dependence on commercial milk formula, which negatively affects both short- and long-term breastfeeding practices.

Pasteurized DHM was also used for older infants, such as abandoned ones, with post-pasteurization tests showing acceptable levels of 10 to less than 100 CFU/mL. This expanded the scope of DHM usage, providing critical nutrition to a broader age group of vulnerable infants.

### Monitoring indications to reduce the duration and volume of DHM usage

In 2020, the Da Nang HMB analyzed trends in DHM usage. To reduce reliance on DHM, the hospital introduced guidelines for prescribing DHM and tracking the availability of mothers’ own milk, alongside enhanced breastfeeding support efforts. The hospital used the Decision Tree for Donor Human Milk designed to help guide the prioritization and allocation of donor human milk, ensuring optimal nutrition and preventing misuse [10]. Consequently, the duration of DHM usage in neonatal and postnatal wards was reduced.

### Changing the protocol for splitting pasteurized DHM

In the initial stage, Da Nang HMB distributed whole containers (e.g., 200 mL each) to neonatal units or postnatal wards, where the staff would defrost and split pasteurized DHM to feed the infants. Data from the monitoring system showed a higher rate of milk disposal because the volume was more than needed, and the defrosted milk had an expiration date. The procedure has since been changed so that HMB staff defrost and split the DHM into portions (each feed for each infant in need) at the HMB. These portions are then sent to neonatal or postnatal units in a cold chain and stored in a fridge before feeding the infants. This approach also helps to better ensure the process and condition of defrosting and splitting DHM. It also helps to better manage other aspects such as label printing and consumables management.

### Scaling up the HMB monitoring system beyond Da Nang HMB

The successful establishment and operation of Da Nang HMB have fostered knowledge exchange and collaborations within Vietnam and across the Southeast Asia region. Alive & Thrive, in collaboration with the Philippines HMB Association, has established the Southeast Asia Human Milk Bank Network to enhance the quality and reach of HMB services in the region. This network promotes regional collaboration, provides technical support, and facilitates knowledge exchange among HMBs. Through webinars, training sessions, and sharing best practices, the network aims to standardize operations and improve the safety and efficiency of milk banks, ensuring more infants benefit from DHM. A significant outcome of this regional association includes the development of regional standards for HMB, which will be discussed later.

**Fig. 6:**
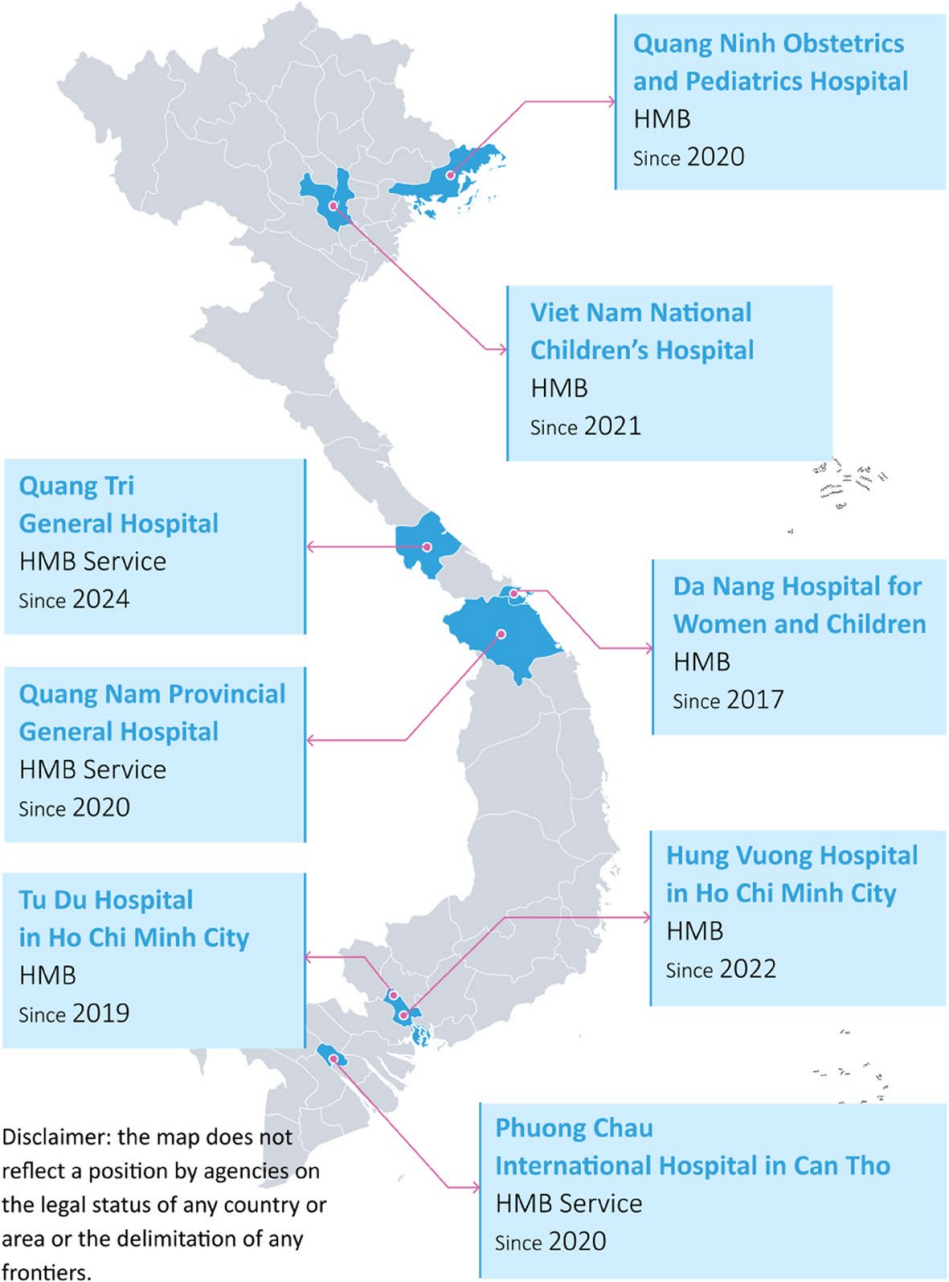
Scale up of the human milk bank services in Vietnam

In Vietnam, since the establishment of Da Nang HMB, HMB service has been scaled up from Da Nang Hospital for Women and Children to four other HMBs and three HMB services (Fig. 6). An HMB service collects and stores raw DHM, sending it through a cold chain to the corresponding HMB for handling and processing. It then receives pasteurized DHM through a cold chain from this HMB and uses it for newborns in need in their hospital [11].

The rollout of the digital monitoring system also extends beyond Da Nang HMB to Tu Du Hospital in Ho Chi Minh City, as the second HMB was established in the country in 2019, using the same digital solution by another local IT firm in Ho Chi Minh City (Y Viet Co.) with technical assistance from PHD Ltd in and Alive & Thrive. The HMB monitoring system in Tu Du Hospital was built on the platform, experience, and expertise gained from developing the monitoring system at Da Nang HMB. The planning stage for Tu Du HMB included understanding the needs and expectations from the outset and agreeing on the scope with the IT companies. Then, most of the communication regarding development, gathering feedback, and implementing improvements was conducted virtually through calls or messaging using Zalo. This platform served as a channel for users to report issues or suggest improvements, allowing Y Viet Co. or other parties to address them promptly.

After approximately one year of operation, the HMB staff work directly with Y Viet Co. to manage, sustain, and improve the monitoring system. The hospital pays a fee per their agreement with the IT company.

New functions have been developed, such as financial reporting and the recording and reporting of consumables (e.g., syringes and containers), which can be counted separately. The updated system also prints out summaries of utilization and receipts by clients, components covered by health insurance, and the remaining balance payments of clients to the hospital. All improvements from Tu Du HMB are then integrated into Da Nang HMB and vice versa. Tu Du HMB has also integrated its HMB monitoring system into the hospital health information system, storing the information on the hospital server. Tu Du Hospital still does not have a digital medical record system to integrate with the HMB monitoring information. However, it would be feasible to link the systems by using a unique client record or national personal identification number.

When the HMB service in Quang Nam was established, the same PHD Ltd and Alive & Thrive partnership supported the creation of its monitoring system, which includes components for raw DHM collection and pasteurized DHM distribution. The monitoring systems of Da Nang HMB and Quang Nam HMB are connected to store and share information about donors, donations, pasteurization, and DHM distribution. Additionally, information about the conditions during transportation and receipts is tracked.

The remaining three HMBs in Vietnam adapted monitoring forms from Da Nang HMB, using the open access code provided by PHD Ltd to develop their monitoring systems. The developers were typically from the hospitals’ IT units or contractors. Despite differences, core indicators are kept the same.

### External dissemination and use of data

#### Research and publications

Data collected from HMB operations were used in various studies, informing different aspects of HMB practices and being published in peer-reviewed journals.

In our efforts to strengthen the health system, we published research on enhancing newborn nutrition through establishing the HMB in Vietnam [7], followed by general guidance on using DHM [3]. Using monitoring data, we analyzed trends and dynamics over the first four years of the HMB’s operation [12]. We examined factors associated with the use of pasteurized DHM for healthy newborns [13] and factors associated with the prolonged use of DHM [14], drawing from the experience of this first HMB in Vietnam.

Regarding DHM donors, we published studies on the characteristics and factors influencing the volume of DHM by women to the first HMB in Vietnam [15]. This includes a case report on experiences and perspectives on bereavement and breastmilk donation [16], as well as views on wet nursing and expressing breastmilk for sharing and HMB donation among mothers [17].

Regarding the operation of this HMB, we published findings on the differences in the microbiological profile of raw and pasteurized breastmilk from hospital and community-based donors [18]. Using an activity-based costing ingredients study, we also conducted a cost analysis of establishing and operating the first HMB at Da Nang Hospital for Women and Children in Vietnam.

#### Costing estimation for the establishment and operation of an HMB

The costing study was conducted to inform hospitals about the startup and operational costs of establishing Da Nang HMB [9]. By analyzing the volume of raw and pasteurized DHM, the study can estimate the cost per liter of DHM and identify factors that affect this unit cost [9]. These studies provided valuable insights for decision-makers considering the implementation of HMBs. Furthermore, based on this costing paper and toolkit, we established a beta version of a global costing tool for establishing and operating HMB [19].

#### Cost and benefit estimations for the national health system

Data from HMB monitoring systems have been used for the Policy Report—Health Insurance Coverage for Pasteurized Donor Human Milk. This report not only highlights the health benefits of Pasteurized DHM for these small vulnerable infants but also underscores the potential economic gains for Viet Nam’s state budget if DHM is covered by social health insurance [20]. This study utilized various information from HMBs in Da Nang and Tu Du, including the number of small vulnerable newborns, the number of newborns in need of DHM, the volume of DHM used, the duration of DHM use, and the costs for the establishment and operation of an HMB, among other factors [9, 20]. This data has been used to advocate for the inclusion of DHM in insurance plans for children, which will help make DHM more accessible to all small vulnerable newborns in need [9, 20].

#### Development of national and regional HMB guidelines

The information gathered was instrumental in developing hospital-specific guidance, National Human Milk Bank Guidelines for Vietnam [11] and the Minimum Standards for the Establishment and Operation of Human Milk Banks in Southeast Asia [21]. These resources ensured standardized practices and improved the quality and safety of HMB services in Vietnam and the region [11, 21]. The national guidelines, including instructions on the HMB monitoring system, were heavily based on the guidelines from HMBs in Da Nang [11]. Drawing from Vietnam’s experience and with support from external experts, regional standards for HMBs [21] have been developed and approved by member states in Southeast Asia. The instructions on the monitoring system were also based on the experience of Vietnam’s HMB monitoring system [11, 21].

## Discussion and lessons learned

### Global platform and importance of HMB monitoring

Currently, there are more than 700 HMBs globally [22]. Despite this widespread presence, there has yet to be a comprehensive study on the status of HMB monitoring systems worldwide. However, based on our knowledge of selected HMBs in East Asia Pacific and Western Europe, each HMB employs its own monitoring system, which can vary significantly. These systems range from app-based or web-based platforms to computer logbooks (e.g., Excel spreadsheets) and hardcopy, paper-based logbooks. The capacities of these systems, including the type and amount of information generated and the associated workload, can also differ widely.

An open access digital monitoring system for HMBs is crucial for several reasons. Firstly, it ensures the efficient storage and management of information, which is vital for the smooth operation of HMBs. By systematically collecting and storing data, these systems provide a comprehensive overview of all activities, from donor screening to milk distribution. This informs daily operations and enriches the knowledge base, allowing for continuous improvement in practices and protocols. Additionally, digital systems enhance transparency, making tracking and tracing donor milk easier, ensuring it meets safety standards, and can be reliably traced back to its source. This is particularly important for maintaining trust and ensuring the safety of the milk provided to vulnerable infants. Digitalization helps to reduce the use of resources such as printers, cartridges, paper, and storage space. It also helps store information for longer durations than paper-based systems and minimizes concerns about the disposal of medical information printed on paper. As our study indicates, digital information can easily and effectively generate knowledge that can be disseminated in different forums.

Moreover, digital monitoring systems align with guidance globally [23, 24] and in Vietnam [25], and trends in digitalization, supporting broader public health goals. They facilitate the tracking and tracing of DHM, essential for maintaining milk kinship and ensuring ethical practices. By integrating these systems, HMBs can provide detailed reports and data analytics, which are invaluable for policymaking and improving public health strategies. This alignment with digitalization initiatives enhances the efficiency and effectiveness of HMBs and ensures they are part of a modern, integrated healthcare system.

### Work opportunities

The HMB monitoring system is designed as a web-based, lightweight platform, ensuring ease of access and efficiency in low-resource settings, including devices and internet connection. The system provides robust and scalable storage solutions by storing data on a server or cloud, facilitating seamless data management and retrieval. This setup allows different units to input data simultaneously, enhancing collaboration and efficiency across various locations. Additionally, storing data in the cloud ensures that it is not lost due to local storage failures, providing a reliable and secure data management solution.

The involvement of a dedicated local IT team ensures that the system is well-maintained and that any technical issues are promptly addressed, enhancing reliability and user experience. Moreover, the system is developed with a forward-thinking approach, encouraging teamwork and discussions to identify the best solutions. This collaborative environment fosters innovation and continuous improvement. The system is also tailored to meet the specific needs of HMBs, ensuring that while it adapts to unique requirements, the core functionalities remain consistent and effective. This balance between customization and stability makes the HMB monitoring system a reliable and future-proof solution.

### Areas for improvements

#### Using the same system for all HMBs in the network

Using the same system for all HMBs in Vietnam's specialized service network can offer several benefits. Although it was the initial plan, implementing a streamlined system across all HMBs would facilitate easy data aggregation from the network. This would enhance tracking, comparison, and research capabilities. To achieve this, each new HMB would purchase a membership and allocate a Level of Effort (LOE) for an IT consultant to tailor the system to their specific needs. This approach also serves as a business model for the IT firm. By centralizing HMB monitoring, we would have the opportunity to ensure maintenance, updates, and technical support beyond any external assistance. The Ministry of Health would also more likely to manage and finance the HMB monitoring system.

However, some HMBs decided not to adopt this system, believing it was simple enough to manage without external support. Additionally, at that time, we needed more financial resources to support the development of this system in other HMBs. As a result, we do not have a unified system for all the facilities in Vietnam, which leads to fragmentation in maintenance, updates, technical support, and financing by each HMB.

Because of the need to gather key performance data from all HMBs, we later supported a parallel system. The HMB staff need to extract the information from their HMB monitoring system to add to this system, which takes additional effort and time. However, this additional effort reduces the chance of having complete data timely from the HMB network.

#### The connection between the Da Nang HMB and the microbiology unit

The connection between the Da Nang HMB and the microbiology unit was also an issue. The process of sending lab requests and receiving results involved paper forms, which required additional time and human resources. The improvement was the introduction of electronic requests from the HMB to the microbiology unit, specifying the timing for sending samples. This change would reduce delays and manual entry errors. Subsequently, lab staff can enter test results directly into the system, allowing real-time updates accessible to HMB staff. However, the lab staff still prefers having paper-based requests and a call notification before sending the sample. They also send the results back to the HMB by paper, and the HMB staff enter the information into the monitoring system.

#### The app-based monitoring system in smart phone or tablet

An app-based system could benefit significantly, especially as it supports temporary offline data storage on local computers where internet connectivity is unavailable. This feature is particularly important in low-resource settings, where internet bandwidth may be limited or interrupted. Additionally, having a compact mobile device for certain activities would be advantageous, especially donor recruitment. The HMB can continue its operations without significant disruptions by ensuring the system remains functional even in these conditions.

We planned to develop an app for iOS and Android devices. However, key challenges include the need for lengthy clearance from the app stores and high annual fees. We decided not to pursue this option. The monitoring system still works well in Safari, MS Edge, and Chrome on any smart device, so it is not a major issue. In some situations, using paper-based forms before entering the information into the web app is also an option. Thus, managing without an app is feasible.

Nonetheless, most of the use of the HMB monitoring system requires a computer screen to work efficiently. This ensures that all functionalities are accessible, and that data entry and management are performed accurately and effectively.

#### Unique ID of DHM containers across HMBs

These IDs are needed to distinguish DHM containers across HMBs, especially when distributing DHM within the country or internationally [4]. ISBT 128 is a global standard for the identification, labeling, and information transfer of medical products of human origin, including human milk. It ensures consistency and safety in handling and tracking these products across different healthcare systems [26]. For human milk banks, ISBT 128 provides a standardized way to label and track milk donations by assigning a globally unique identifier to each donation, which helps maintain traceability and ensure the safety and quality of the milk. This standard also facilitates the integration of milk banking systems with other healthcare systems, improving overall efficiency and reliability [26]. However, there are costs involved in using ISBT 128. Organizations must pay an initial one-time registration fee and an annual licensing fee, which can be expensive and cumbersome for hospitals [26]. Having extensive information in the system and labels also incurs costs related to upgrading the system and utilities (cartridges and paper). Additionally, to make it useful, many hospitals need to register and retain the license with ISBT 128 [26].

Only a few hospitals have HMBs or their services in Vietnam, and they are typically in different provinces and regions, so the demand for complex tracking across HMBs is not needed for now. An interim step of assigning a hospital code could be manageable. However, changes are required in the coding and labeling system. We decided not to pursue this at the current stage because the distribution level beyond the HMB or its services is minimal. For DHM containers being sent out, we have an additional label to indicate the HMB source and destination.

## Conclusions and recommendations

The establishment of the first HMB in Vietnam at the Da Nang Hospital for Women and Children has been a significant milestone in improving neonatal care in Vietnam, which is crucial for the health of infants when the mother's own milk is unavailable. The application of a locally developed and open access digital monitoring system has ensured the smooth operation, quantity, quality, and safety of DHM. The system's features, including donor management, DHM processing, and recipient management, have streamlined operations and enhanced efficiency. The data management system has been instrumental in improving program performance, informing decisions, supporting research, and developing national and regional guidelines and standards. The successful scaling up of HMB services and the monitoring system to other regions in Vietnam demonstrates the project's effectiveness and potential for broader impact. Learning from the experience of the Da Nang HMB can also aid in developing HMB monitoring systems for other HMBs in Vietnam and other countries worldwide.

## Supplementary Information

Additional file 1. Paper-based monitoring forms.

Additional file 2. Information technology (IT) platform and design of the human milk bank monitoring system.

Additional file 3. Human milk bank monitoring system - User manual (only available in Viet-namese).

## Data Availability

No datasets were generated or analysed during the current study.

## Abbreviations

DHM: Donor human milk
DMADV: Define, Measure, Analyze, Design, and Verify
EENC: Early Essential Newborn Care
HMB: Human Milk Bank
KMC: Kangaroo mother care
LBW: Low birthweight
SOPs: Standard Operating Procedures
