# Supplementary material for: Application of a locally developed open-access digital monitoring system for the human milk bank network in Vietnam
Source: Int Breastfeed J. 2025 Jul 8;20:54. doi: 10.1186/s13006-025-00745-1 (PMC12239282; doi:10.1186/s13006-025-00745-1)
Supplement: Supplementary file 1 — Additional file 1. Paper-based monitoring forms. [file 13006_2025_745_MOESM1_ESM.pdf]

APPENDIX 1. SOP 19. Monitoring and Reporting

Human Milk Bank project at Da Nang Hospital for Women and Children

1. Purposes

1.1. To provide timely routine monitoring data to optimize the functionality of the Human Milk Bank (HMB).

1.2. To ensure that all activities of the HMB meet standardized protocols. Ensure the tracking and tracing of donor human milk.

1.3. Provide data for studies (e.g., cost, effectiveness) and the formation of the National Guidelines on HMB. Data from all indicators and research will be used for this purpose.

2. Overview

- Alive & Thrive Vietnam in partnership with PATH, the Ministry of Health (Maternal and Child Health Department) and the Da Nang Department of Health aims to establish the first Human Milk Bank (HMB) in Vietnam in the Da Nang Hospital for Women and Children to ensure access to lifesaving human milk to save newborn lives.
- **Figure 1** summarizes the flow of process practices of this HMB.
- The monitoring system included 12 monitoring forms (**Table 1**): A Monthly Report (BC 1); 4 forms for donors (BM 1 – BM 4), 3 forms for HMB (NH 1 – HN 3), and 4 forms for recipients and Neonatal units (e.g., intensive care units and postnatal units; KH 1 – KH 4). **Appendix 3** includes forms and instructions. The number of forms will be less when the the electronic system is fully implemented.
- Monitoring data are gathered, managed and used mainly by HMB staff with the support of the HMB manager and staff of A&T and PATH. Update schedule is found in Table 1.
- Real-time data and monthly reports are generated for program improvement and tracking of progress of HMB indicators.

Figure 1. Flow of process practices in human milk banking

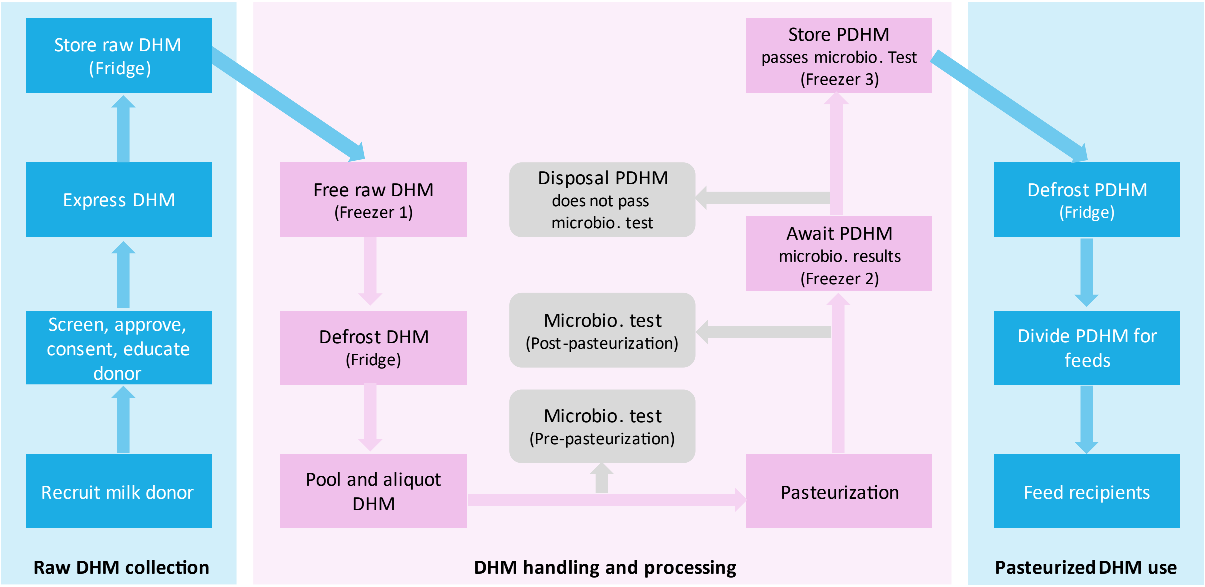

Table 1. Summary of monitoring forms

The list	Update schedule	Place	Related operating procedures and purpose	Web-based function
BC 1. Monthly Report	Monthly	HMB	Reporting. Information for BC 1 was from BM 1 & 4; NH 1; and KH 2 & 3.	Reports and Charts
BM 1. Demand generation logbook	Ongoing	HMB, Neo- & postnatal units	Donor recruitment	Demand generation
BM 2. Registration of Human Milk Donors	Ongoing	HMB, Neo- & postnatal units	Registering potential donor<br>Screening and checking for eligibility	No
BM 3. Donor record	Ongoing	HMB	Screening, participation consent, HMB approval for participant, donor education, donor health update, and at the time of stopping milk donation.	Donor record
BM 4. Milk donation logbook	Ongoing	HMB	Donation of milk for each mother	Milk donation record
NH 1. Milk tracking log sheets at pasteurization room	Ongoing	HMB	Monitor the amount of donor milk at various steps at HMB: receiving, storage, defrosting, pasteurization, distribution and disposal of donor milk.	No, can be generated through Mild donation record and Pasteurization record
NH 2. Pasteurization record	Ongoing	HMB	Defrosting, pooling, pasteurization, laboratory test, pasteurization, and approval of pasteurized donor human milk (PDHM). Pasteurization process should be logged and attach to this form.	Pasteurization record
NH 3. Distribution logbook	Ongoing	HMB	Distribution and transportation of PDHM from HMB to neonatal units and postnatal wards	Distribution record
KH 1. Consent for a feeding of Pasteurized donor human milk	Ongoing	Neo- & postnatal units	Consent for the feeding of PDHM (a legal document)	(No, info link with User record)
KH 2. User record	Ongoing	Neo- & postnatal units	Feeding record of PDHM by each child in neonatal units (amount of PDHM consumed and the amount of money the family will pay)	User record
KH 3. Container tracking logbook	Ongoing	Neo- & postnatal units	Use of PDHM in neonatal units: tracking each container to verity if it follows SOPs, estimate the amount of PDHM used, and provide 2-way tracking from donors to users.	Container tracking record
KH 4. Donor milk usage log sheet	Ongoing	Neo- & postnatal units	Usage of PDHM in neonatal units: to help nurses stock appropriate amount of PDHM for following shifts.	Donor milk usage record

Appendix 3.

BC 1. Monthly Report - Báo cáo tháng

From date….. month………year 20…. To date….. month………year 20….

**1. Demand Generation and Donor Recruitment**	**Total**	**In the hospital**	**Community**	**Other**
1.1. Number of group counseling sessions for demand generation (≤ 10 mothers)				
1.2. Number of demand generation events (> 10 mothers)				
1.3. Number of lactating mothers who attended donor recruitment sessions (total):				
1.3.1. One-on-one sessions				
1.3.2. Group sessions				
1.3.3. Events				
1.4. Number of mothers who expressed interest in donating after recruitment				
1.5. Number of mothers newly screened				
1.6. Number of lactating mothers newly screened and meeting all eligibility requirements to become human milk donors				
1.7. Number of eligible human milk donors trained in proper hygiene and donation skills (e.g., handwashing, safe milk expression, storage, labeling, and transport to the HMB)				
1.8. Number of new donors who donated milk				
1.9. Number of mothers who stopped donating milk (e.g., due to child age above 9 months, disease status, personal decision, or absence of milk donation for 90 days at this HMB)				
**2. Receiving, Storage, Processing, and Distribution of Donor Milk**				
2.1. Volume of milk donated (L)				
2.2. Volume of pasteurized donor human milk (DHM) (L):				
2.2.1. Passed pre-pasteurization test (L)				
2.2.2. Passed post-pasteurization test (L)				
2.2.3. Passed both pre- and post-pasteurization tests (L)				
2.3. Volume of donor milk disposed of for any reason (L)				
2.4. Total volume of pasteurized DHM available at the HMB at the reporting time (L)				
**3. Usage of Pasteurized DHM**		**Neonatal units**	**Postnatal wards**	**Other**
3.1. Volume of distributed pasteurized DHM (L)				
3.2. Number of children who started receiving pasteurized DHM from the HMB				
3.3. Number of children currently receiving milk from the HMB				
3.4. Volume of pasteurized DHM fed to infants (L)				
3.5. Average number of days pasteurized DHM was used (for infants who stopped receiving DHM during the reporting period)				
3.6. Amount of money received (thousand VND)				

BM 1. Demand generation logbook - Sổ ghi chép vận động hiến tặng

**Date**<br><br>(1)	**Duration** (minutes)<br><br>(2)	**HMB donor recruiter**<br>(3)	**Location**			**No. of participants by method**			**No. of potential donors**<br>(10)
			HMB<br>(4)	Neonatal<br>(5)	Other (specify)<br>(6)	Inter-personal<br>(7)	Group<br>(8)	Event<br>(9)	

Instructions: For each demand generation activity, fill in the date, time, and person completing the event (Columns 1-3); mark location (columns 4-6) and the number of people that participated in: column 7 for individual, column 8 for group, and column 9 for “other” type event. From the initial verbal agreement of the mothers, fill in the number of potential donors in column 10 and fill in the list in BM 2.

By the end of the month, fill the Total row for columns 4-10 and report the number to HMB staff (if from other departments). HMB staff combine the number for the whole hospital to fill in the monthly report.

BM 2. Registration of Human Milk Donors - Phiếu đăng ký sàng lọc bà mẹ (Can be done directly to BM 3)

Unit: 1. HMB; 2. Neonatal ………..; 3. Postnatal ………..; 4. Other (specify) ……………….

Registration (Columns 1-3): ……/……/ 201….. Responsible person:…………………………….

Checking (Columns 4-14): ……/……/ 201….. Responsible person:…………………………….

**Name of potential donor**<br><br>**Year of birth**<br><br>**Contact phone number**	**Address**	**Gave birth at DNHWC**	**Had screening lab test results**				**Evaluate lab test**		**The next step**			**Interview and lab test arranged**	**Screened**
			HIV	HBV	HCV	Syphilis	≥ 1 test (+)	> 6 months	Lab test + Screen. interview	Screen. interview	Not qualify		
(1)	(2)	(3)	(4)	(5)	(6)	(7)	(8)	(9)	(10)	(11)	(12)	(13)	(14)

Instructions: For this table, fill 1 for Yes, and 0 for No. Might bypass this form by filling in directly BM 3.

1) Initially, ask each mother about a "Positive" or "Abnormal" results of any Antenatal test results. If there is a positive result, inform the mother that she might not eligible, refer her to appropriate counseling, and do not list her name on this form.
2) If the test results for the mother are "Negative" or "Normal", the procedure will vary depending on the location:

Postnatal Ward: Fill columns 1-3; check medical record to fill in columns 4-9, and send the form to HMB

Neonatal Units or HMB: Fill columns 1-3 for all potential donors, write people in charge and date and send to HMB (if from Neonatal Units); check medical records (for those who delivered at Da Nang Hospital for Women and Children) and medical tests from other hospital (for those who delivered elsewhere to fill column 4-9.

3) Fill columns 10-12, and set up appointments with potential donors at HMB for donor screening.

BM 3. Donor record - Hồ sơ bà mẹ hiến tặng

DONOR SCREENING FORM (circulate to corresponding number of fill in the blank)

Interview date: ……/……/ 201…..

Names:…………………………………….. Year of birth: ……………..

Age:…………….; if < 18 years, stop interviewing

Address: No. ………., street/village:………..……………. ward/commune:…………………….……………..

District:……………………., city/province ……………….Contact phone: …………..……………

Child date of birth: ……/……/ 201….. Birth place: 1) Da Nang Hospital for Women & Children

No. of gestation weeks: ………. week 2) Other……………….……………….

Birth weight: ………. grams Mode of delivery: *1. Vaginal 2. Cesarean section*

From whom did you hear about donating breast milk? (multiple-choice question)

1. Staff of Human Milk Bank
2. Staff at Obstetric Unit (antenatal care)
3. Staff at Postnatal Unit
4. Staff at Neonatal Unit
5. Other mothers or any other
9. Have not yet heard about donating

Where else did you hear about donating breast milk? (multiple-choice question)

1. Poster
2. Leaflet
3. Newspapers, magazines
4. Events
5. Facebook (Fanpage)
6. Website / internet
7. Video clip
8. Other……………….……………….
9. Not any

I will ask you some questions. All information will be secured kept and only used for the selection of milk donors.

	Question	No	Yes
1.	Have you received a blood transfusion transplant in the last 6 months?	0	1
2.	Have you ever had hepatitis B or C, TB or cancers?	0	1
3.	Are you taking medication, traditional medication, substance, chemical, radio-active drugs? If yes, please list:	0	1
4.	Have you had a Measles, Mumps and Rubella (MMR) vaccine in the 4 weeks?	0	1
5.	Do you smoke or using any products with nicotine such as patches, lozenges, sprays containing nicotine; nicotine gum; the electronic cigarette?	0	1
6.	Do you regularly have more than 2 alcoholic drink weekly? An alcoholic drink is equal to 1/2 brandy (25ml), a glass of wine (100ml), or a cup of beer (200ml).	0	1
7.	Have you ever used one of substances / drugs such as opium, marijuana, cocaine, heroin, crystal meth, ecstasy?	0	1
8.	In the last 6 months have you participated in unsafe sex (not using condom) with your husband or partner who is at risk for HIV, HBV, HCV, or syphilis?	0	1
9.	In the last 6 months Have you pierced any part of your body or gotten a tattoo or scaring?	0	1
Comments of observation from screening staff (if any):			

Lab test (within 3 months before the screening):

[ ] Test for HIV, HBV, HCV and syphilis negative [ ] Print or photocopy the test results

[ ] Eligible to give consent; ___________________________________(name of staff performing the screening)

INFORMED CONSENT TO BE A HUMAN MILK DONOR

(To be read and signed by mother)

Human milk provides the best nutrition for infants and young children. It promotes comprehensive growth and development and helps to reduce the risk of illness. Human milk has a unique composition of nutrients, enzymes, growth factors, anti-inflammatory and immune properties. **The best option for a baby is its mothers own milk.**

When this is not available, **the next best option is pasteurized donor human milk from a milk bank**. Pasteurized milk is **safe, doesn’t contain any food preservatives** and **retains most of** the nutrients, growth factors, anti-inflammatory and immune properties.

I confirm that:

1. I am 18 years of age or older.
2. I am donating excess / surplus breast milk after feeding my own baby. I am committed to having a healthy lifestyle during breastfeeding and donation (for example, I do not drink alcohol, or smoke and/or use illegal drug).
3. I understand that my donated human milk will be stored, processed and microbiologically tested to ensure it safe use in vulnerable infants. A responsible doctor will prescribe human milk for a recipient in need of donor milk as regulated by the human milk bank.
4. I will not expect my donated human milk to be returned to me or to receive compensation for my donated human milk.
5. I have the right to stop donating breastmilk at any time and will not be treated differently when I or my child use the hospital health care or services.
6. I undertake to inform the human milk bank of any changes in my health as soon as I become aware of it. Human milk bank telephone number is 05113 957 177.
7. I understand that all my personal information, including medical test results will be kept confidential.

I hereby give consent for my donated breast milk to be given by the human milk bank of the Da Nang hospital for Women and Children to any infant who are hospitalized and unable to access to their own mother milk. I also give consent for using my demographic information and donation for statistics and research to improve the human milk bank’s operations and to create knowledge.

CONSENT	Da Nang……/……/ 201…..<br>Donor name	 HMB staff
ATTENDING THE DONOR EDUCATION SESSION	……/……/ 201…..	
APPROVAL	……/……/ 201…..	
DONOR ID		
DATE STOPPED DONATING	……/……/ 201…..	

BM 4. Milk donation logbook - Sổ theo dõi hoạt động hiến tặng sữa (this form can be omitted in the electronic system)

**ID_Do**	**Donor information**			**Date of**			**Information about each donation** (fill in the date donated in the first row and the number of mL in second row. Information about expression date will be written in the label)									
	Name	Year of birth	Contact phone	Screen ing	Approval	Training	1	2	3	4	5	6	7	8	…	30
(1)	(2)	(3)	(4)	(5)	(6)	(7)										

Column 1-7 fill in information from BM 3. Each mother will have 3 rows. Fill information about each donation time (i.e., send milk to the HMB), horizontally. If used all 30 cells, fill in the next row.

NH 1. Milk tracking log sheets at pasteurization room - Phiếu theo dõi lượng sữa trong tủ của phòng thanh trùng (Hang the form on appropriate freezers or fridge. In the electronic system, it will be fill automatically by scanning container ID).

Location (circle appropriate code):

NH 1.1. Freezer 1 (Raw donor milk)	HN 1.2. Fridge (Defrosting)	HN 1.3. Freezer 2 (Wait for lab test)	HN 1.4. Freezer 3 (Ready for distribution)

Month ……/ 201…..

**Date**	**Amount in** (mL)	**Amount out for use** (mL) (i.e., change location or distribution)	**Unused amount** (mL) (e.g., disposal, loss)	**Amount remain** (mL)
(1)	(2)	(3)	(4)	(5)
0				
1				
...				
31				
**Sum:**				

Row at date "0", fill the amount of milk remaining from the previous month:
Column 2-4, if ≥ 2 events a day, fill separately for each time separating by plus sign (+): e.g., 100 + 200.

NH 2. Pasteurization record - Hồ sơ loạt thanh trùng

**A. DEFROST**	Time; date	Fridge temperature	Implementer
**Start:**	……h……min; ……/……/ 201…		
**End:**	……h……min; ……/……/ 201…		

Information about defrosting of donor human milk:

Donor ID	Name	No. of containers	Amount (mL)	Expired date
1.				
2.				
3.				
4.				
5.				

B. POOLING, POURING TO CONTAINER FOR PASTEURIZATION

**Start**	……h……min; ……/……/ 201…	**Status of implementation:**<br>[ ] Fully defrosted<br>[ ] In the hood

**C. LAB TESTS**	No. of sample	Sample ID
Pre- Pasteurization		[ ] print test result
Post-Pasteurization		[ ] print test result

D.PASTEURIZATION Time: start: ________________ end: _______________ [ ] print Pasteur. chart

Batch No:…….……. Pool No.:……. Total milk: ………. containers; ………….mL

Implementer: Signature_________________ Names____________________________ Date……/……/ 201…

APPROVAL

Donor ID	Container number	Amount (mL)	Approver
**Meet requirements:**			Đà Nẵng ……/……/ 201…<br><br>Signature______________________<br><br>Name_________________________<br><br>Comments (if any):
**For disposal** (did not meet requirements):			

NH 3. Distribution logbook for pasteurized donor human milk - Sổ xuất sữa từ ngân hàng sữa mẹ (HMB) (Printed in A3 logbook. In the electronic system this form can be combined with NH 1.4. with electronic signature, then this form can be reduced)

Month ……/ 201…

Date (1)	Neonatal units			Other (mostly postnatal units, if other, specify)		
	Container ID (ID_Bo) (2)	Volume (mL) (3)	Recipient signature (4)	Container ID (ID_Bo) (5)	Volume (mL) (6)	Recipient signature (7)
1						
2						
...						
31						

KH 1. Informed consent for receipt of donor human milk

Recipient unit: 1) Neonates care ward…………; 2) Post-natal unit……………….; 3) Other……………….

Human Milk provides the best nutrition for infants and young children. It promotes comprehensive growth and development and helps to reduce the risk of illness. Human milk has a unique composition of nutrients, enzymes, growth factors, anti-inflammatory and immune properties. **The best option for a baby is its mothers own milk.**

When this is not available, **the next best option is pasteurized donor human milk from a milk bank**. Pasteurized milk is **safe, doesn't contain any food preservatives** and **retains most of** the nutrients, growth factors, anti-inflammatory and immune properties.

My name is ………………………………………… the mother (or primary caregiver) of

………………………………………… infant. I understand that:

1. Donated human milk has been **stored, processed and microbiologically tested** at the discretion of the human milk bank, Da Nang Hospital for Women and Children.
2. Donated human milk is **safe to be used for the newborn**, including low birth weight, preterm, and sick infants. Responsible doctor will prescribe human milk for recipient as regulated by the Da Nang hospital for Women and Children.
3. Human milk donors have met all criteria to be safe human milk donors. They are all healthy, have healthy lifestyle (do not drink alcohol, smoke and use illegal drugs), have undergone medical tests and be confirmed that they are free of any infection with HIV, hepatitis B, hepatitis C and Syphilis. All human donors' personal information, including health condition and medical test results will be kept confidential.
4. Stock of donor milk may be limited and not always available for all infants.
5. The hospital will charge a fee of using donated human milk to cover part of operation costs. The fee is approved by Da Nang City Department of Health.
6. I will accept lactation support to ensure that I can supply sufficient milk to my infant.
7. I/ recipient mother need to establish my own supply of breast milk so will attempt to feed my own baby or express 8-12 times in 24 hours
8. I confirm I am 18 years of age or older. (If mother is under 18 years old, the infant legal guardian will sign).

After explained by my infant doctor, I agree to use donated human milk for my infant and to pay for the fee as the hospital regulation. I also give consent for using my child's demographic and clinical information and the use of donor human milk for statistics and research to improve the human milk bank's operations and to create knowledge.

Đà Nẵng ……/……/ 201…

Recipient mother or legal guardian name: Medical officer

______________________________ ______________________________

KH 2. Record of users of donor human milk - Phiếu tổng hợp thông tin liên quan tới trẻ nhận sữa

- For each child receiving PDHM, fill in 1-12, 15, 16 based on the medical record and stick the form to the medical record. Fill in the information of the child to computer to link with the utilization of PDHM (forms KH 3 and KH 4).
- Every day, information will be updated automatically from forms KH 3 and KH 4 to the second page of this form.
- Alternatively, fill in the amount of PDHM used from form KH 4 to corresponding line in page 2 of KH 2. Computer will calculate items 18-20 when by the date fill in item 17.

Unit: 2. Neonatal ………..; 3. Postnatal ………..; 4. Other (specify) ……………….

1. Med. record ID:…………………………….. 2. Child name:…………………………….

3. DOB: ……/……/ 201….. 4. Sex: *1. Boy* *2. Girl*

5. Mother's name:………………………………. 6. Father's name: …………………………………..

7. Address: No. ………, street/village:………..……………. ward/commune:…………………….………

District……………………., City/Province ……………….Phone No.: …………..……………

9. Delivery mode: *1. Vaginal* *2. Cesarean section*

	Date	Diagnose / status
11. Hospitalized	……/……/ 201…	
12. Unit admission	……/……/ 201…	
13. Unit discharge	……/……/ 201…	
14. Hosp. discharge	……/……/ 201…	

Information relating to using of pasteurized donor human milk

15. Indication of physician (multiple choices):

Relating to the child	Relating to the mother
1. Low birth weight <1,500gr with disease(s)<br>2. Preterm born <32 weeks with disease(s)<br>3. Serious disease (list)<br>4. Preterm or low birth weight without disease (non-disease)<br>5. Full-term whose mother used to donate milk<br>6. Full-term without disease<br>7. Under 6 months with special disease (cancer, immune deficiency, heart disease, gastrointestinal disease)	1. Mother unable to express sufficient milk<br>2. Mother unavailable (e.g., dead, abandoned child)<br>3. Having drug treatment with contraindication for breast feeding<br>4. Has no milk in the first few days after birth because of any reasons.

16. Start date: ……/……/ 201…; 17. End date: ……/……/ 201…

Summary about the use (from the following page; calculated automatically for electronic system):

18. No. of days (Count column 2): ………….. days

19. Volume consumed (Count column 3): ………….. mL

20. Expenditure on donor milk (Count column 4): ………………….. VND

Implementer	Verifier	Data entry
Date……/……/ 201…	Date……/……/ 201…	Date ……/……/ 201…

Amount of pasteurized donor human milk usage

(one copy in medical record, and the other for mother to keep and update)

1. Med. record ID:……………………….. 2. Child name:…………………………….

5. Mother name:……………………………… 3. DOB: ……/……/ 201… 4. Sex: *1. Boy* *2. Girl*

No<br>(1)	Date<br>(2)	Amount (mL)<br>(3)	Expenditure (VND)<br>(4)
1			
2			
3			
4			
5			
6			
7			
8			
9			
10			
11			
12			
13			
14			
15			
16			
17			
18			
19			
20			
21			
22			
23			
24			
25			

No	Date	Amount (mL)	Expenditure (VND)
26			
27			
28			
29			
30			
31			
32			
33			
34			
35			
36			
37			
38			
39			
40			
41			
42			
43			
44			
45			
46			
47			
48			
49			
50			

KH 3. Container tracking logbook - Sổ theo dõi từng chai sữa mẹ hiến tặng thanh trùng tại khoa

Unit: 2. Neonatal ………..; 3. Postnatal ………..; 4. Other (specify) ……………….

**ID_Bo**	**Receipt**		**Defrost**		**Open**		Amount after defrost (mL)	Used: 1. All 0. Not all	Amount disposed (mL)	**Name child-mother** (separate by semicolon)
	Date	Time	Date	Time	Date	Time				
(1)	(2)	(3)	(4)	(5)	(6)	(7)	(8)	(9)	(10)	(11)

For paper based system: For each container, fill in information in columns 1-8. Column (9), fill 1 if used all, and 0 if did not (e.g., need to throw away because > 24 after completely defrosted) and fill in the amount did not use of this container to column (10). For every child received milk from the container, fill in child-mother name. At a specific time of the day, write the date in the ID_Bo cell, and write sum the total amount after defrost (Column 8) and amount disposed (column 10) and estimate amount used = column (8) – (10).
For electronic system: Information will be tracked using ID_Bo. The name of child-mother of each unit will be popped up when scan the container for use.

KH 4. Pasteurized Donor Human Milk Use log sheet - Theo dõi sử dụng sữa mẹ hiến tặng thanh trùng tại khoa

Unit: 2. Neonatal ………..; 3. Postnatal ………..; 4. Other (specify) …………….. Date: ……/……/ 201…

**Child name**	**Mother name**	**Room, bed**	**Indication**		**Verify**	**Time to feed**												Total amount
			No. of meals	mL / meal		7-8	9-10	11-12	13-14	15-16	17-18	19-20	21-22	23-24	1-2	3-4	5-6	
(1)	(2)	(3)	(4)	(5)	(6)													(8)
					BM Suff[1]													
					mL													
					BM Suff													
					mL													
					BM Suff													
					mL													
					BM Suff													
					mL													
					BM Suff													
					mL													
					BM Suff													
					mL													
					BM Suff													
					mL													

This form will be used at postnatal or neonatal units to support the Nurses to estimate the amount of milk to take each time. From the indication in the medical record, fill columns 1-5 for each child. At feeding time, nurses ask the mothers about the amount she could express. If breastmilk sufficient (BM suf.), fill 1, and leave blank cell "mL". If not, fill the amount of PDHM. At the beginning of the day (e.g., 7 AM) sum horizontally the amount of PDHM to fill column 8 and corresponding row of KH 2 for each child.
