# Supplementary material for: Application of a locally developed open-access digital monitoring system for the human milk bank network in Vietnam
Source: Int Breastfeed J. 2025 Jul 8;20:54. doi: 10.1186/s13006-025-00745-1 (PMC12239282; doi:10.1186/s13006-025-00745-1)

Annex 2. IT platform and design of the HMB monitoring system

Scope of Work: Develop and maintain the electronic monitoring system of the first Human Milk Bank in Da Nang Hospital for Women and Children

1. **Background**

Alive & Thrive Vietnam in partnership with PATH, the Ministry of Health (Maternal and Child Health Department) and the Da Nang Department of Health aims to establish the first Human Milk Bank (HMB) in Vietnam in the Da Nang Hospital for Women and Children to ensure access to lifesaving human milk to save newborn lives.

One of the key components of the HMB project are the monitoring system. The purposes of the system are to 1) provide timely routine monitoring data to optimize the functionality of the Human Milk Bank (HMB), ensure that all activities of the HMB meet standardized protocols and ensure the tracking and tracing of donated human milk; and 3) provide data for studies (e.g., cost, effectiveness) and the formation of the National Guidelines on HMB.

The monitoring system included 12 monitoring forms: A Monthly Report (BC 1); 4 forms for donors (BM 1 – BM 4), 3 forms for HMB (NH 1 – HN 3), and 4 forms for clients and Neonatal units (e.g., intensive care units and postnatal units; KH 1 – KH 4). Monitoring data are gathered, managed and used by mainly by HMB staff with the support of the HMB manager and staff of A&T and PATH. Every month, the information is integrated into a Monthly Report to share with HMB management board, A&T and PATH. Data from different months will be gathered to project the trend. For a more detail description about the monitoring form.

To collect, manage, analyze, and use data more efficiently and timely (i.e., lower burden for health staff, verify and analyze data quicker, tracing the donated milk sample, estimating the amount of donated milk at different locations of the donated milk chain), an electronic system is needed. We envision that the HMB monitoring system include 1) online database; 2) computer program, and 3) android and Apple applications.

2. Flow of process practices in human milk banking

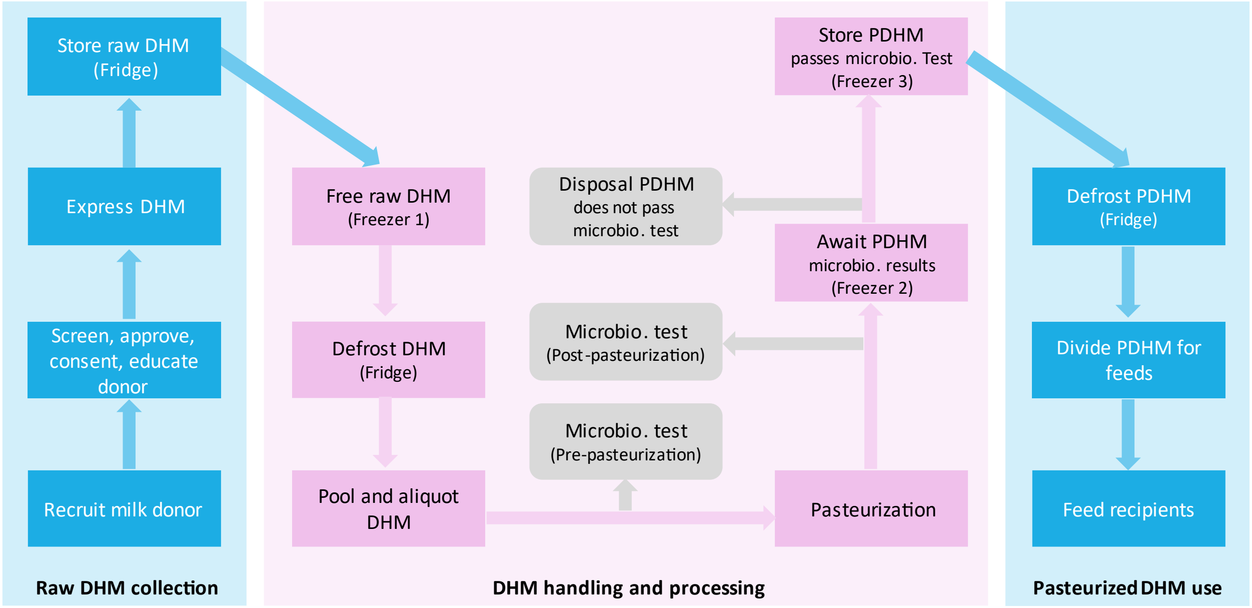

3. Main functions

No.	Function name	Description
**A**	**Web application**	
1	Demand generation logbook	- Input demand generation activity
2	Donor record management	- Input donor information<br>- Input daily milk donation
3	Pasteurization record management	- Input pasteurization information<br>- Print bottles' labels
4	Distribution logbook for pasteurized donor human milk	- Input distribution information
5	Record of users of donor human milk	- Input users of donor human milk information<br>- Manage daily expense
6	Bottle tracking logbook	- Input used bottles daily, including who use them
7	Pasteurized Donor Human Milk Use log sheet	- Input the daily amount of milk take by users
8	Equipment management	- Manage information of equipment used by HMB center including: general information, maintenance cycle
9	Periodic report	- Extract information from any reporting cycle
10	Export raw data	- Extract raw data from database for later uses
11	Other reports	- Reports required during use
12	Expiry date alert for donor human milk	- Alert functions in home screen for expiry date
**B**	**Mobile app**	
1	- Input donor record<br>- Tracking bottle information by barcode<br>- Input defrosted time/open time/consumed volume of pasteurization milk	

4. General database structure

Platform: Microsoft SQL Server 2008 R2

Database relation

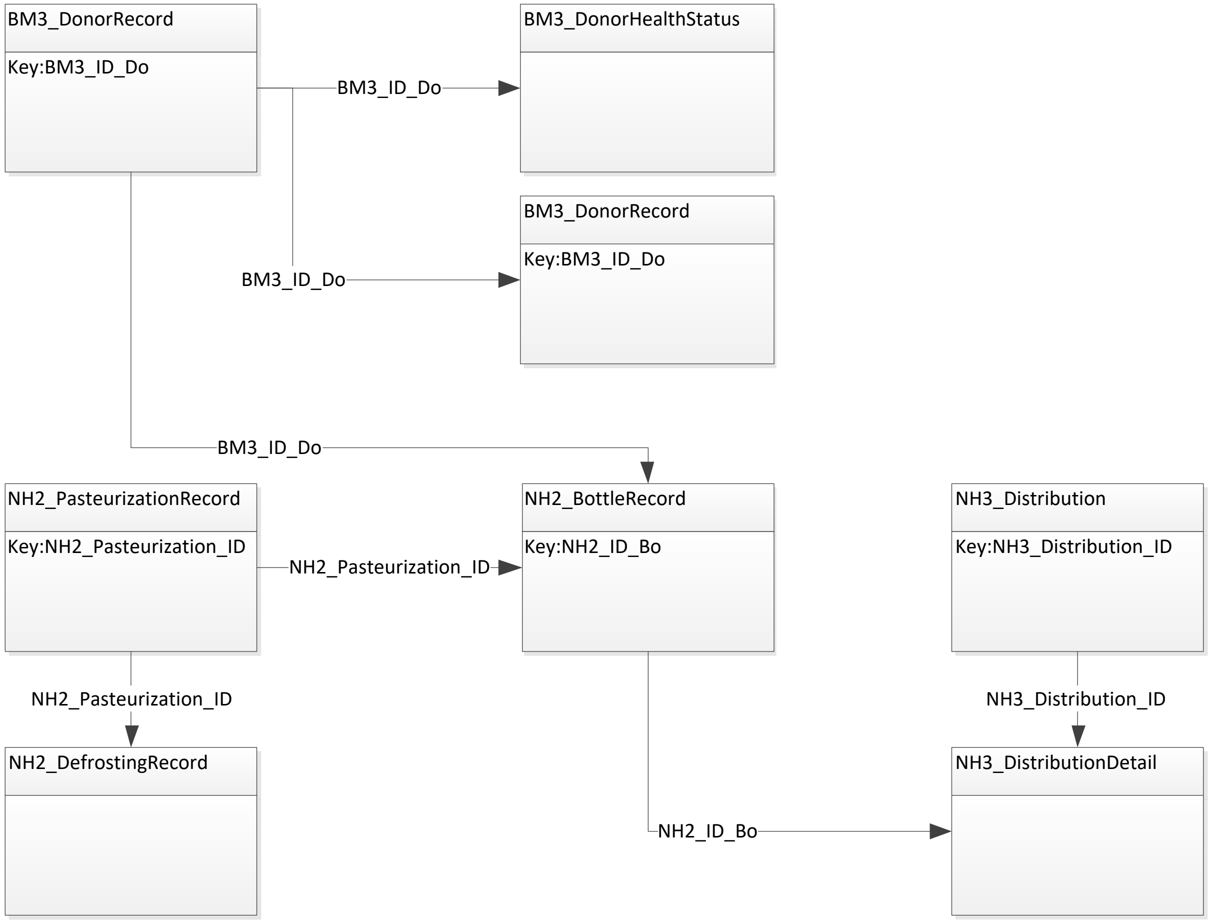

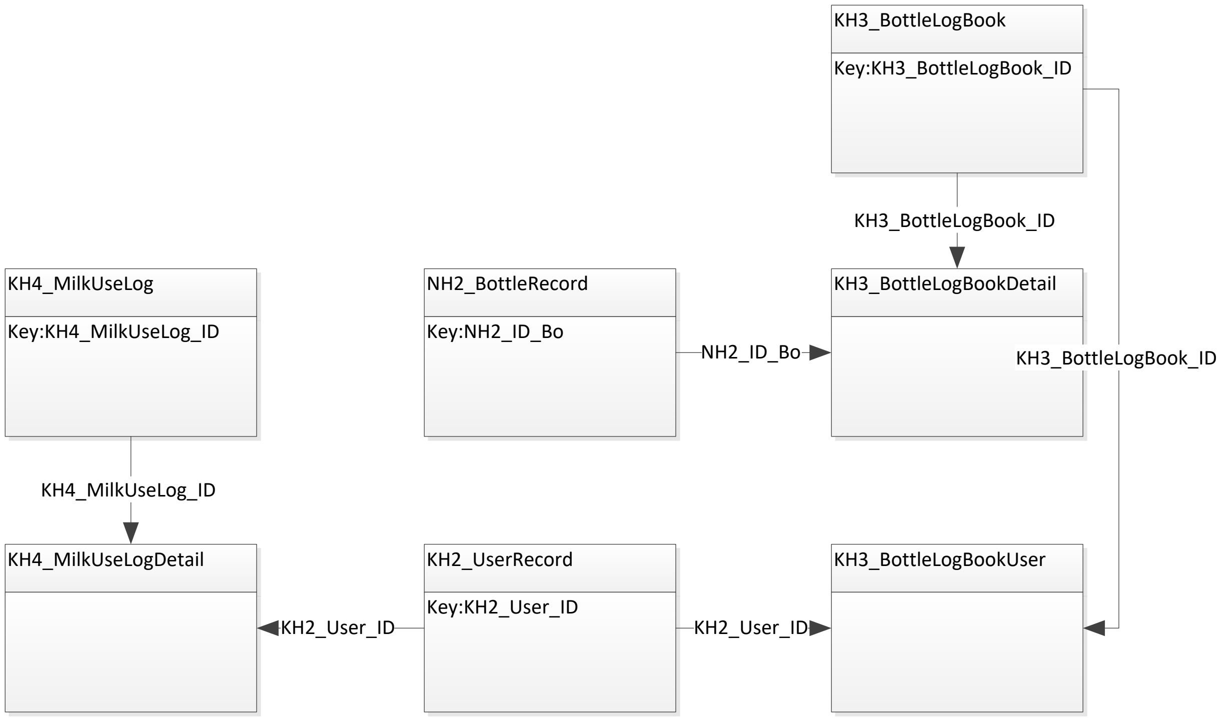
KH3_BottleLogBook
Key:KH3_BottleLogBook_ID
KH3_BottleLogBook_ID
KH4_MilkUseLog
Key:KH4_MilkUseLog_ID
NH2_BottleRecord
Key:NH2_ID_Bo
NH2_ID_Bo
KH3_BottleLogBookDetail
KH3_BottleLogBook_ID
KH4_MilkUseLog_ID
KH4_MilkUseLogDetail
KH2_User_ID
KH2_UserRecord
Key:KH2_User_ID
KH2_User_ID
KH3_BottleLogBookUser

Function flow vs. database

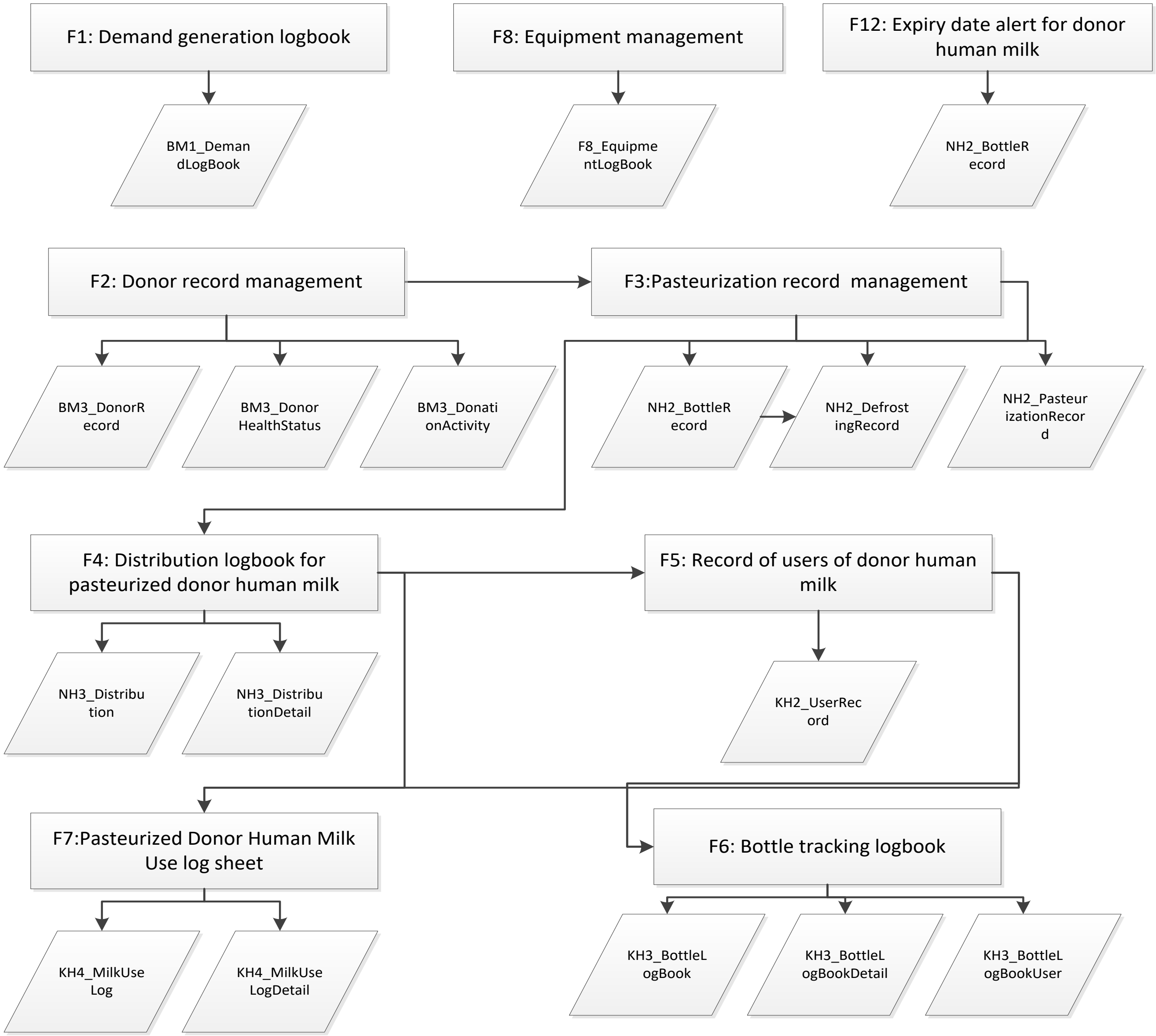

Supplement: Supplementary file 2 — Additional file 2. Information technology (IT) platform and design of the human milk bank monitoring system. [file 13006_2025_745_MOESM2_ESM.pdf]
