# Supplementary material for: Application of a locally developed open-access digital monitoring system for the human milk bank network in Vietnam
Source: Int Breastfeed J. 2025 Jul 8;20:54. doi: 10.1186/s13006-025-00745-1 (PMC12239282; doi:10.1186/s13006-025-00745-1)
Supplement: Supplementary file 3 — Additional file 3. Human milk bank monitoring system - User manual (only available in Viet-namese). [file 13006_2025_745_MOESM3_ESM.pdf]

KẾT NỐI PHẦN MỀM QUẢN LÝ NGÂN HÀNG SỮA MẸ TỪ DŨ

Table of Contents

Thông tin chung 2
Xác nhận phần mềm đã được gắn kết vào hệ thống thông tin bệnh viện 2
Cửa sổ đăng nhập vào phần mềm Ngân hàng sữa mẹ tại server của Bệnh viện Từ Dũ 2
Quản lý mật khẩu và đăng nhập 3
Quản lý người sử dụng và quyền truy cập phần mềm 3
Danh sách người sử dụng 3
Thêm mới người sử dụng 3
Quản lý quyền truy cập 3
Sàng lọc và thu nhận sữa mẹ 4
Theo dõi vận động hiến tặng 4
Quản lý bà mẹ hiến tặng 4
Cửa sổ chung 4
Quản lý những lần hiến tặng sữa của từng bà mẹ 4
Hồ sơ bà mẹ hiến tặng: Chọn bà mẹ hiến tặng nội viện 5
Hồ sơ bà mẹ hiến tặng: Chọn bà mẹ hiến tặng ngoại viện 5
Quản lý hoạt động tại Ngân hàng sữa mẹ 6
Quản lý các khâu của hoạt động thanh trùng 6
Quản lý chai sữa sau thanh trùng ở các giai đoạn khác nhau 7
Cửa số chính 7
Quản lý chai sữa ở giai đoạn cấp đông 7
Rã đông sữa để đưa vào sử dụng 7
Quản lý chai sữa trong quá trình rã đông 7
Quản lý và sử dụng sữa mẹ thanh trùng 8
Quản lý trẻ nhận sữa 8
Cửa sổ chính 8
Đăng ký trẻ nhận sữa 8
Order và thống kê lượng sữa sử dụng tại khoa 9
Order sữa từ khoa 9
Tạo order mới 9
Kiểm tra các order cũ 9
Ghi nhận và thống kê lượng sữa và vật tư tiêu hao của từng trẻ hàng ngày 9
Tổng kết kết thúc sử dụng 10
Báo cáo tổng hợp hoạt động Ngân hàng sữa mẹ 11
Báo cáo hoạt động 11
Báo cáo vật tư tiêu hao 12

Thông tin chung

Xác nhận phần mềm đã được gắn kết vào hệ thống thông tin bệnh viện

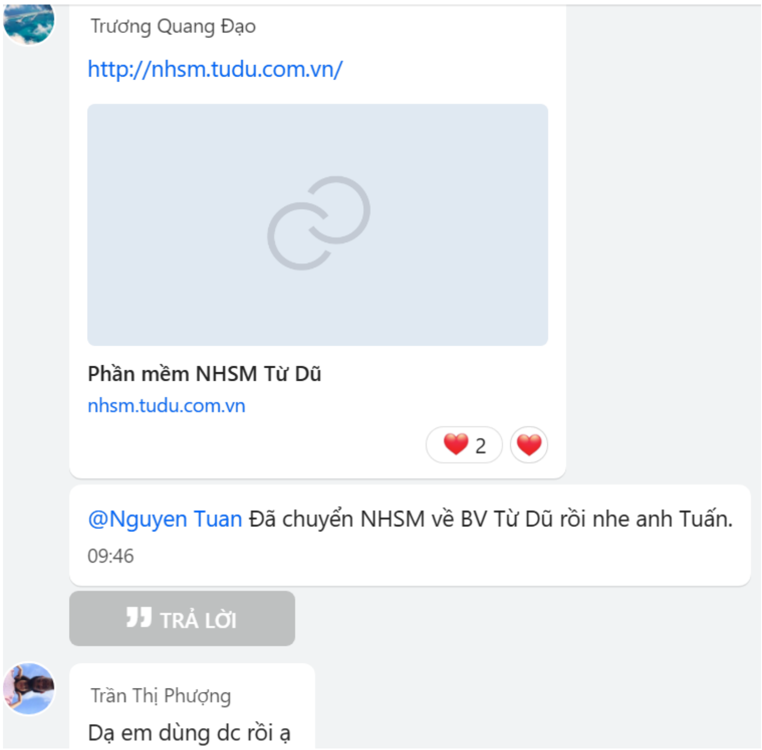

Cửa sổ đăng nhập vào phần mềm Ngân hàng sữa mẹ tại server của Bệnh viện Từ Dũ

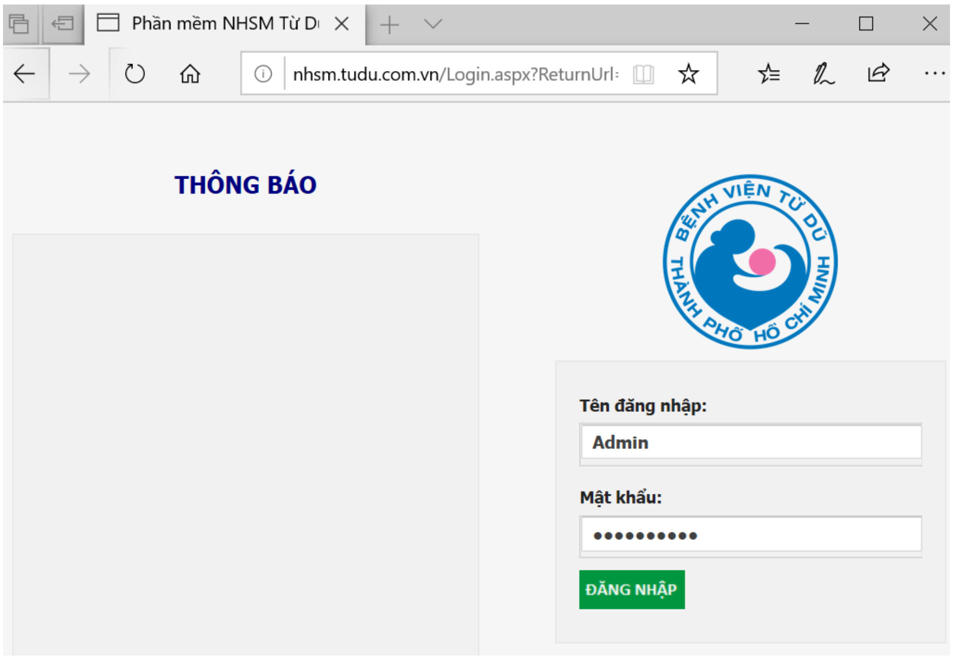

Quản lý mật khẩu và đăng nhập

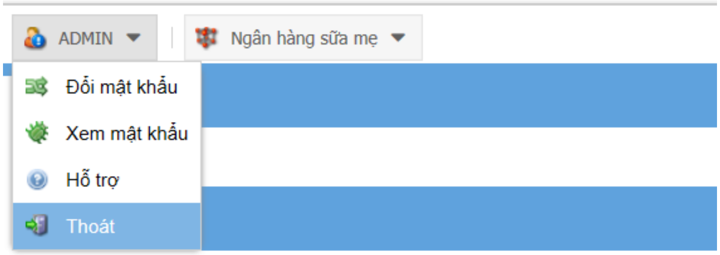

Quản lý người sử dụng và quyền truy cập phần mềm

Danh sách người sử dụng

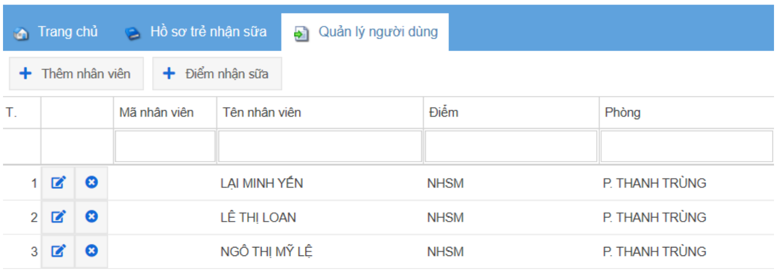

Thêm mới người sử dụng

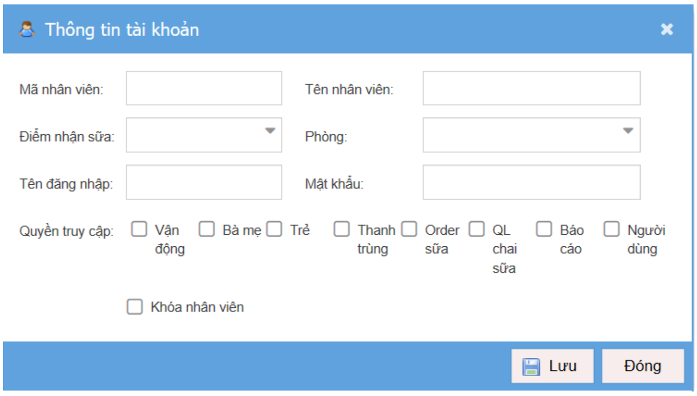

Quản lý quyền truy cập

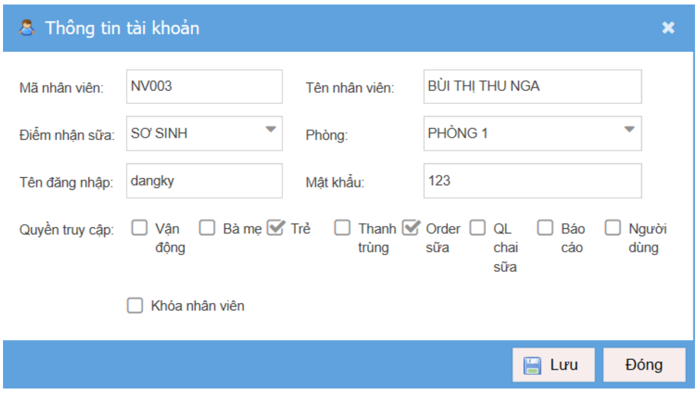

Sàng lọc và thu nhận sữa mẹ

Theo dõi vận động hiến tặng

Trang chủ | Vận động hiến tặng | Hồ sơ bà mẹ hiến tặng

Ngày	Thời gian (phút)	Tê...	Địa điểm	Tên địa điểm khác	Hình thức	Số người tư vấn	Số người tiềm năng
13/03/2020	45	PH...	Trệt N		Cá nhân	3	0
13/03/2020	20	LAI ...	Trệt N		Cá nhân	2	0
12/03/2020	30	PH...	Trệt N		Cá nhân	2	1
11/03/2020	15	PH...	Trệt N		Cá nhân	1	0
11/03/2020	60	CA...	Nhi Sơ Sinh		Nhóm	4	0
10/03/2020	60	CA...	Nhi Sơ Sinh		Nhóm	5	0
09/03/2020	60	CA...	Nhi Sơ Sinh		Nhóm	4	1
06/03/2020	60	CA...	Nhi Sơ Sinh		Nhóm	4	0
10/03/2020	15	LAI ...	Trệt N		Cá nhân	1	0
10/03/2020	45	PH...	Trệt N		Cá nhân	3	0

Quản lý bà mẹ hiến tặng

Cửa sổ chung

ADMIN | Ngân hàng sữa mẹ

Trang chủ | Vận động hiến tặng | Hồ sơ bà mẹ hiến tặng

Từ: đến: Tình trạng: Đang hiến tặng Mã BM: Tên BM: Tìm + Tạo mới

	Tình trạng	Nơi nhận sữa	Ngày phê duyệt	Mã bà mẹ	Họ và tên	Năm sinh	Ngày sinh con	Quận / Huyện
+	Đang hiến tặng	Cộng đồng	18/03/2020	M20-030	Trần Thị Ngọc Thủy	1979	10/03/2020	Bình Chánh
+	Đang hiến tặng	Cộng đồng	18/03/2020	M20-029	Khuất Hồng Vân	1990	13/12/2019	Quận 2
+	Đang hiến tặng	Bệnh viện	16/03/2020	M20-028	Lâm Thị Phương Uyên	1989	14/02/2020	Tỉnh Biên
+	Đang hiến tặng	Cộng đồng	12/03/2020	M20-027	Đặng Thị Ái Kiều	1985	02/02/2020	Tân Bình
+	Đang hiến tặng	Cộng đồng	12/03/2020	M20-026	Trần Thị Trúc Ly	1992	29/11/2019	Quận 10
+	Đang hiến tặng	Cộng đồng	10/03/2020	M20-025	Dương Thúy Loan	1987	21/11/2019	Quận 4
+	Đang hiến tặng	Cộng đồng	07/03/2020	M20-024	Đinh Thị Nhị Lương	1989	04/02/2020	Quận 9
+	Đang hiến tặng	Bệnh viện	09/03/2020	M20-023	Cù Nguyễn Thanh Phụng	1990	13/02/2020	Xuân Lộc

Quản lý những lần hiến tặng sữa của từng bà mẹ

Lượng sữa chưa thanh trùng: 16800 mL và đã thanh trùng: 13010 mL

T.		Ngày vắt	Ngày thu gom	Số lượng (ml)	Lọ 130ml	Lọ 250ml	Tình trạng	Mã loạt	TT
1	⊗			0					false
2	⊗	12/03/2020	20/03/2020	5750		25	Chưa thanh trùng		false
3	⊗	02/03/2020	12/03/2020	4370		19	Chưa thanh trùng		false
4	⊗	20/02/2020	02/03/2020	6680		24	Chưa thanh trùng		false
5	⊗	29/01/2020	18/02/2020	8630			Đã thanh trùng	1-20086	true
6	⊗	15/12/2019	13/02/2020	4380			Đã thanh trùng	2-20060	true

Hồ sơ bà mẹ hiến tặng: Chọn bà mẹ hiến tặng nội viện

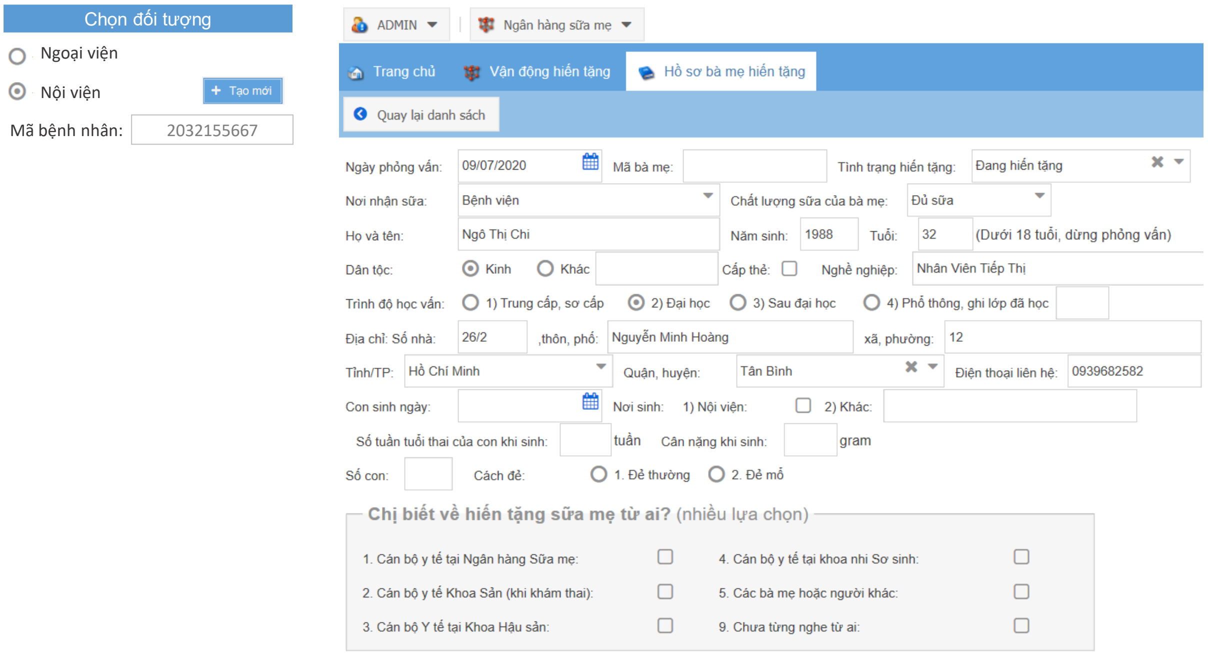

Hồ sơ bà mẹ hiến tặng: Chọn bà mẹ hiến tặng ngoại viện

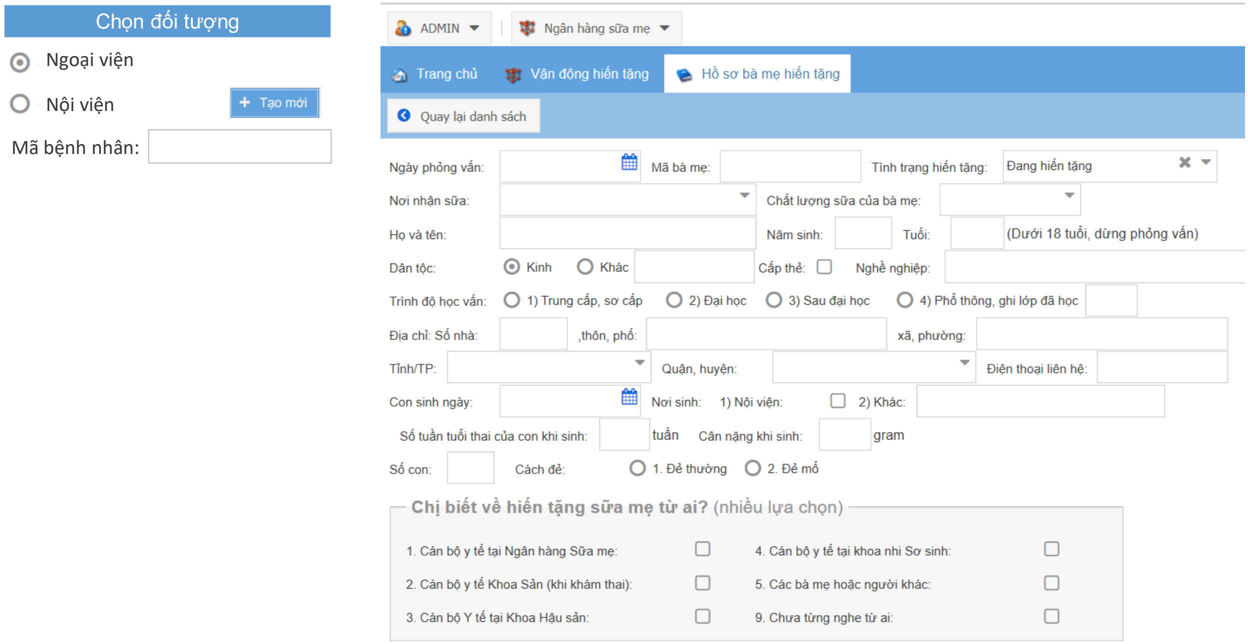

Quản lý hoạt động tại Ngân hàng sữa mẹ

Quản lý các khâu của hoạt động thanh trùng

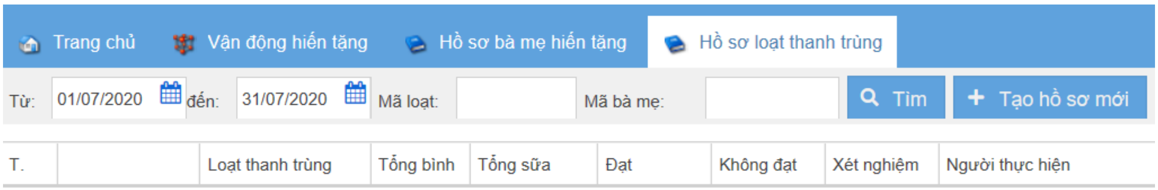

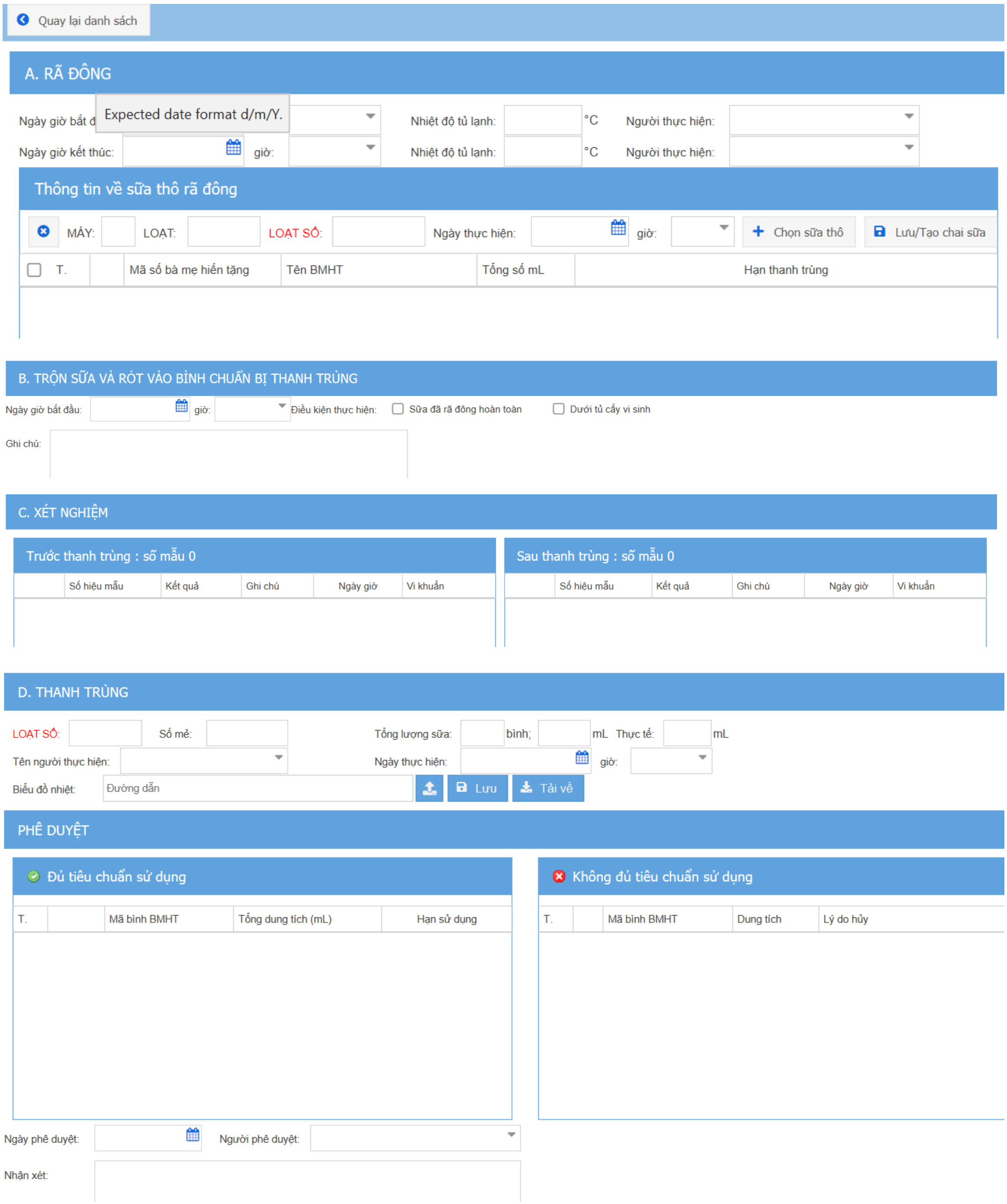

Quản lý chai sữa sau thanh trùng ở các giai đoạn khác nhau

Cửa số chính

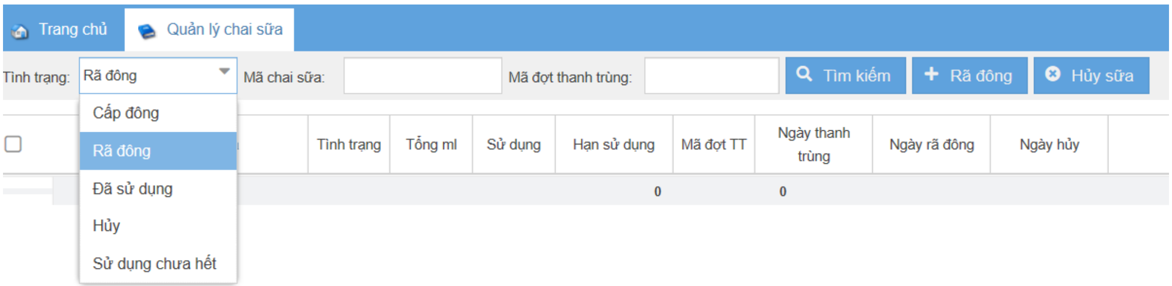

Quản lý chai sữa ở giai đoạn cấp đông

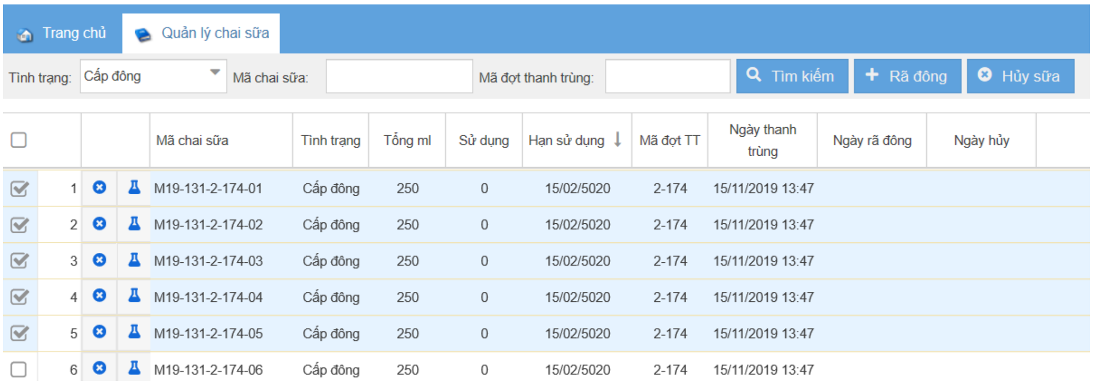

Rã đông sữa để đưa vào sử dụng

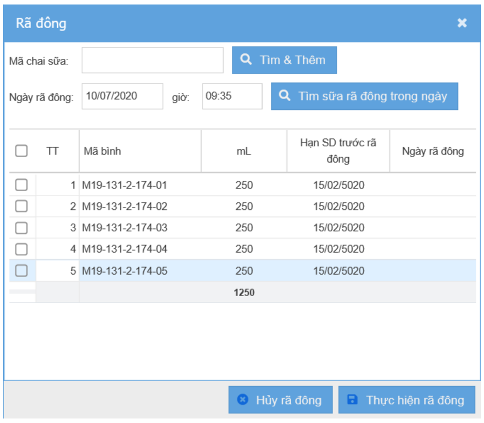

Quản lý chai sữa trong quá trình rã đông

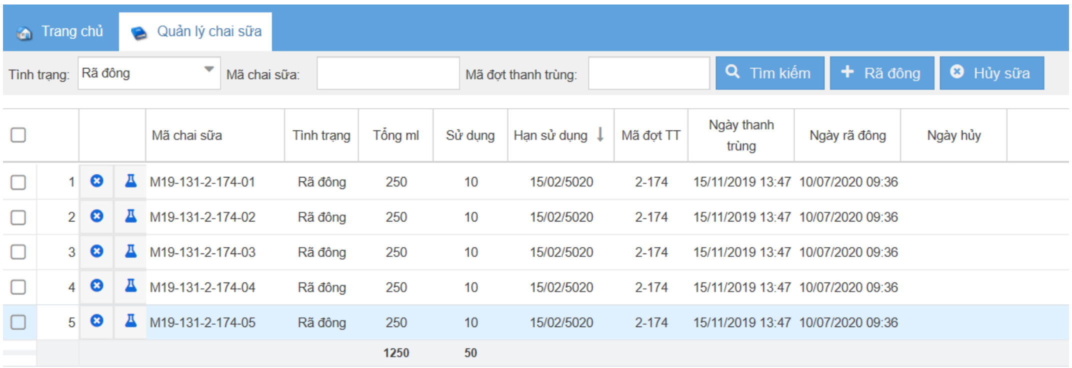

Quản lý và sử dụng sữa mẹ thanh trùng

Quản lý trẻ nhận sữa

Cửa sổ chính

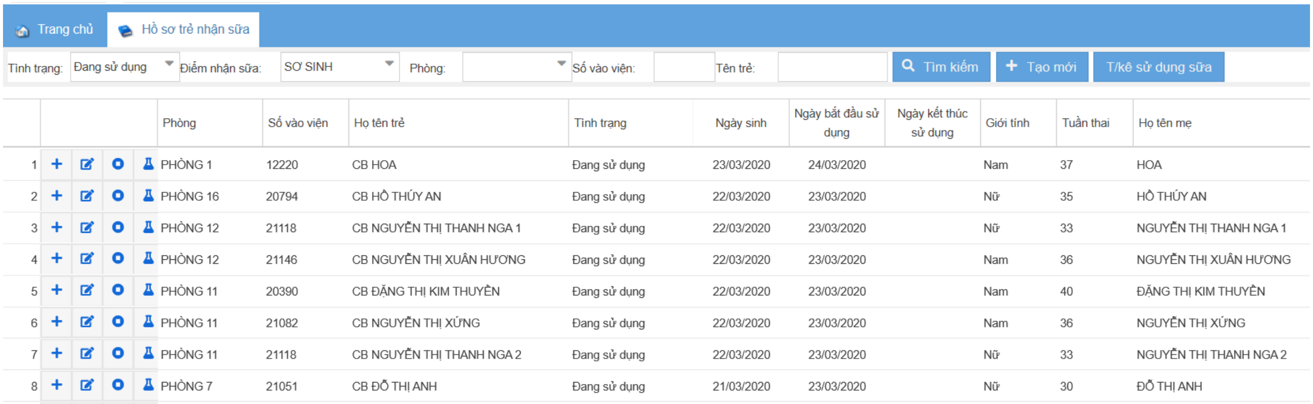

	Phòng	Số vào viện	Họ tên trẻ	Tình trạng	Ngày sinh	Ngày bắt đầu sử dụng	Ngày kết thúc sử dụng	Giới tính	Tuần thai	Họ tên mẹ
1	PHÒNG 1	12220	CB HOA	Đang sử dụng	23/03/2020	24/03/2020		Nam	37	HOA
2	PHÒNG 16	20794	CB HỒ THỦY AN	Đang sử dụng	22/03/2020	23/03/2020		Nữ	35	HỒ THỦY AN
3	PHÒNG 12	21118	CB NGUYỄN THỊ THANH NGA 1	Đang sử dụng	22/03/2020	23/03/2020		Nữ	33	NGUYỄN THỊ THANH NGA 1
4	PHÒNG 12	21146	CB NGUYỄN THỊ XUÂN HƯƠNG	Đang sử dụng	22/03/2020	23/03/2020		Nam	36	NGUYỄN THỊ XUÂN HƯƠNG
5	PHÒNG 11	20390	CB ĐẶNG THỊ KIM THUYỀN	Đang sử dụng	22/03/2020	23/03/2020		Nam	40	ĐẶNG THỊ KIM THUYỀN
6	PHÒNG 11	21082	CB NGUYỄN THỊ XỨNG	Đang sử dụng	22/03/2020	23/03/2020		Nam	36	NGUYỄN THỊ XỨNG
7	PHÒNG 11	21118	CB NGUYỄN THỊ THANH NGA 2	Đang sử dụng	22/03/2020	23/03/2020		Nữ	33	NGUYỄN THỊ THANH NGA 2
8	PHÒNG 7	21051	CB ĐỖ THỊ ANH	Đang sử dụng	21/03/2020	23/03/2020		Nữ	30	ĐỖ THỊ ANH

Đăng ký trẻ nhận sữa

Nội viện

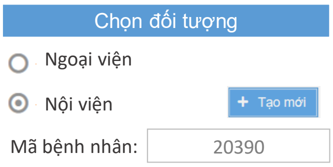

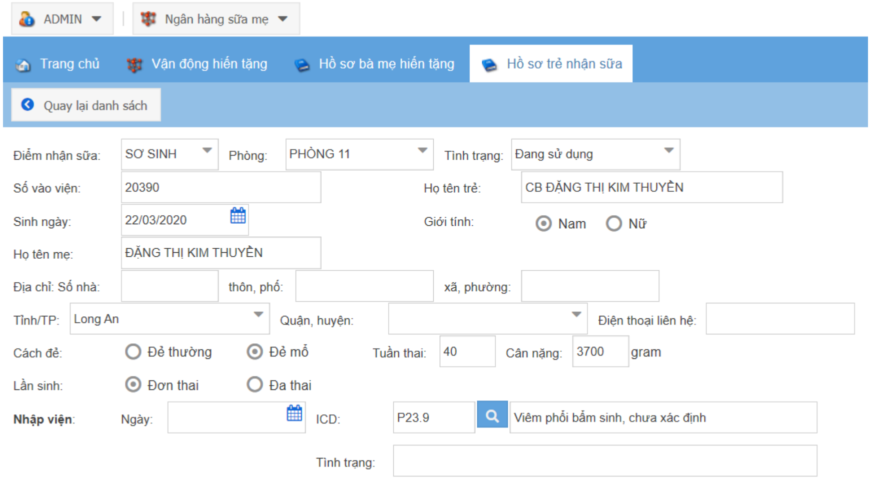

Ngoại viện

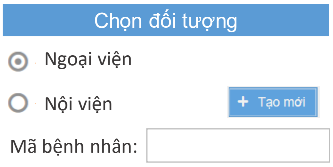

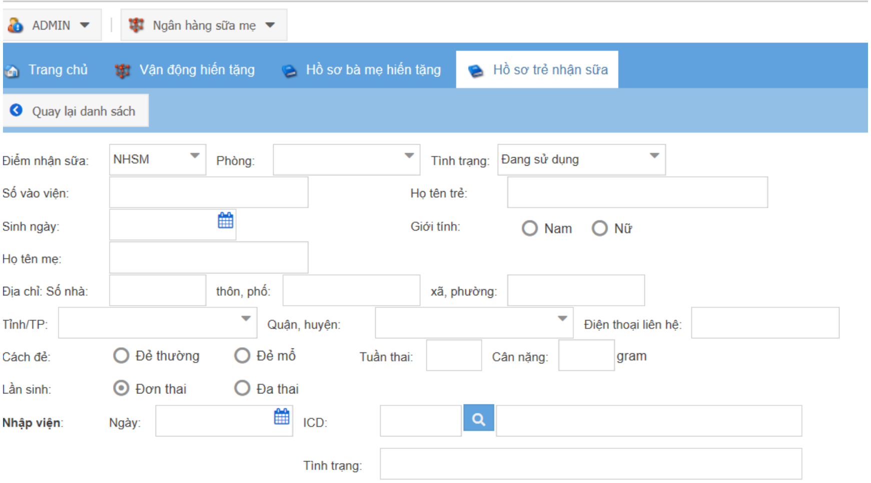

Order và thống kê lượng sữa sử dụng tại khoa

Order sữa từ khoa

Trang chủ | Quản lý chai sữa | Hồ sơ trẻ nhận sữa | Tổng hợp order sữa

Điểm nhận sữa: SƠ SINH | Phòng: | Ngày: 10/07/2020 | Tìm | Tạo order mới | Cấp sữa cho Điểm nhận

T.			Điểm nhận sữa	Phòng	Ngày	Tổng ml sáng	Tổng ml chiều

Tạo order mới

Trang chủ | Quản lý chai sữa | Hồ sơ trẻ nhận sữa | Tổng hợp order sữa

Quay lại danh sách

Điểm nhận sữa: SƠ SINH | Phòng: PHÒNG 3 | Ngày: 10/07/2020 | Người thực hiện:

☐ Khóa sáng ☐ Khóa chiều | Thêm hồ sơ | Lưu

			Phòng	Số NV	Tên trẻ	Sáng từ 7h							Khung giờ sáng			Chiều từ 11h					
☐						mL	Cữ	Tổng		T/thái	Mã chai sữa	Cấp	10h	13h	16h	mL	Cữ	Tổng		T/thái	Mã chai sữa
☐	⊗	1	PHÒNG 3	18938	CB NGUYỄN THỊ HẬU	0	3	0	Ống	Bình thường		0	☑	☑	☑	0	5	0	Ống	Bình thường	
☐	⊗	2	PHÒNG 3	18944	CB TRẦN THỊ THƯƠNG	0	3	0	Ống	Bình thường		0	☑	☑	☑	0	5	0	Ống	Bình thường	
☐	⊗	3	PHÒNG 3	19226	CB TRẦN THỊ TRÚC LINH	0	3	0	Ống	Bình thường		0	☑	☑	☑	0	5	0	Ống	Bình thường	
☐	⊗	4	PHÒNG 3	19807	CB NGUYỄN THỊ THANH THẢO	0	3	0	Ống	Bình thường		0	☑	☑	☑	0	5	0	Ống	Bình thường	

Kiểm tra các order cũ

Trang chủ | Quản lý chai sữa | Hồ sơ trẻ nhận sữa | Tổng hợp order sữa

Quay lại danh sách

Điểm nhận sữa: SƠ SINH | Phòng: PHÒNG 11 | Ngày: 14/01/2020 | Người thực hiện: THU

☑ Khóa sáng ☑ Khóa chiều | Thêm hồ sơ | Lưu

			Phòng	Số NV	Tên trẻ	Sáng từ 7h							Khung giờ sáng		
☐						mL	Cữ	Tổng		T/thái	Mã chai sữa	Cấp	10h	13h	16h
☐	⊗	1	PHÒNG 11	1295	CB TRẦN THỊ HOA	20	3	60	Bú	Bình thường	M19-097-1-190-09/23, M19-092-2-180-25/37	60	☑	☑	☑
☐	⊗	2	PHÒNG 11	2077	CB TRẦN THỊ NGỌC LINH	20	3	60	Bú	Bình thường	M19-112-2-185-09/47, M19-112-2-185-05/13	60	☑	☑	☑
☐	⊗	3	PHÒNG 11	2761	CB HỒ THỊ MAI	25	3	75	Bú	Bình thường	M19-097-1-190-09/75	75	☑	☑	☑

Ghi nhận và thống kê lượng sữa và vật tư tiêu hao của từng trẻ hàng ngày

Trang chủ | Hồ sơ trẻ nhận sữa

Điểm nhận sữa: SƠ SINH | Tên trẻ: CB NGUYỄN THỊ KIM QUYÊN

	Ngày	Sáng		Chiều		TC(mL)	OT 5mL	OT 10mL	OT 20mL(O)	OT 20mL(B)	OT 50mL
		mL/cữ	Số cữ	mL/cữ	Số cữ						
1	23/03/2020	4	3	4	5	32	8	0	0	0	0
2	22/03/2020	4	3	4	5	32	8	0	0	0	0
3	21/03/2020	2	3	2	5	16	8	0	0	0	0
4	20/03/2020	5	3	5	5	40	8	0	0	0	0
5	19/03/2020	4	3	4	5	32	8	0	0	0	0
6	18/03/2020	2	3	2	5	16	8	0	0	0	0

Tổng kết kết thúc sử dụng

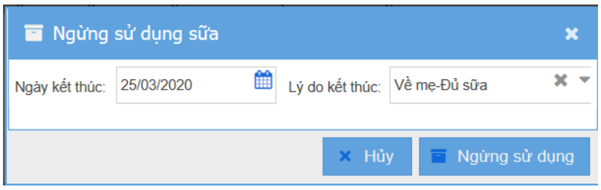

BẢNG CÔNG KHAI SỬ DỤNG

Điểm:SƠ SINH
Phòng:PHÒNG 7

Tên BN:CB NGUYỄN THỊ KIM QUYÊN,Số vào viện:19850
Ngày ngưng sử dụng:25/03/2020,Lý do:Về mẹ-Đủ sữa

**Ngày**	**Sáng**		**Chiều**		**Tổng cộng (mL)**	**OT 5mL**	**OT 10 mL**	**OT 20 mL**		**OT 50mL**
	**SL mỗi cữ (mL)**	**Số cữ**	**SL mỗi cữ (mL)**	**Số cữ**				**O**	**B**	
18/03/2020	2	3	2	5	16	8	0	0	0	0
19/03/2020	4	3	4	5	32	8	0	0	0	0
20/03/2020	5	3	5	5	40	8	0	0	0	0
21/03/2020	2	3	2	5	16	8	0	0	0	0
22/03/2020	4	3	4	5	32	8	0	0	0	0
23/03/2020	4	3	4	5	32	8	0	0	0	0
**Tổng cộng (mL)**					**168**	**48**	**0**	**0**	**0**	**0**
OT BHYT chi trả						10	0	0	0	0
OT Tự chi trả						38	0	0		0

Thân nhân bệnh nhi

Người lập bảng

Báo cáo tổng hợp hoạt động Ngân hàng sữa mẹ

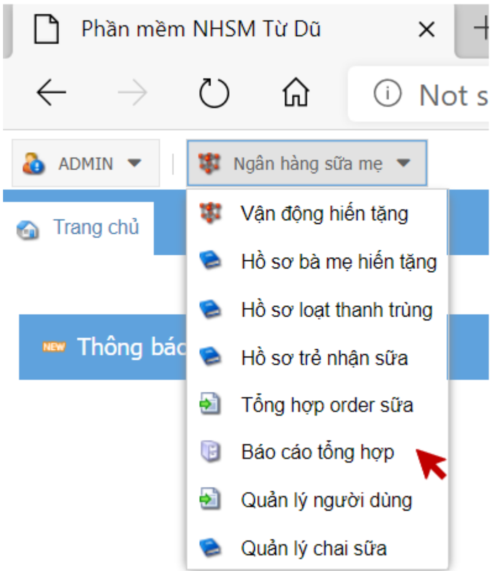

Báo cáo hoạt động

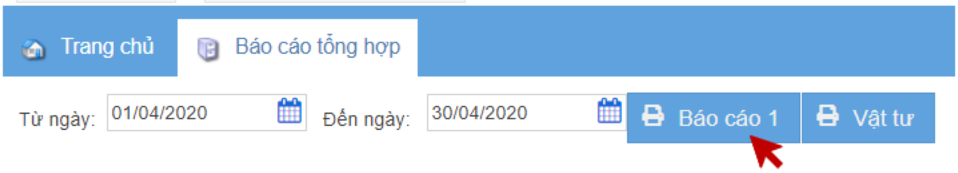

BÁO CÁO TỔNG HỢP Từ ngày: 1/4/2020 đến ngày: 30/4/2020

1. BÀ MẸ HIẾN TẶNG	Vận động tư vấn	94	bà mẹ
	Số bà mẹ tiềm năng	21	bà mẹ
	Số bà mẹ hiến tặng mới	10	bà mẹ
2. THU NHẬN SỮA THÔ	Số lượt tình nguyện viên thu nhận sữa		lượt
	Số lượt nhân viên NHSM tới nhà bà mẹ (lấy máu + CC dụng cụ + thu nhận sữa)		lượt
	SM thô thu gom (l)	369	lít
3. THANH TRÙNG	SM thanh trùng (l)	416	lít
	Số loạt thanh trùng	49	lượt
	Số lượng SM thanh trùng đạt	390	lít
	Số lượng SM thanh trùng không đạt	25	lít
	Tỷ lệ hủy sữa	6.01	%
	SL SMTT không đạt trước TT	14	lít
	SL SMTT không đạt sau TT	0	lít
	SL SMTT không đạt (trước + sau) TT	25	lít
	SL SM thanh trùng đợi KQ XN	0	lít
4. SỬ DỤNG SỮA	Số trẻ sử dụng SMTT (nicu)	878	trẻ
	Số ngày sử dụng trung bình (nicu)	3.0	ngày
	SM SMTT sử dụng (l)	326	lít
	Số trẻ sử dụng SMTT (HS-HP)	3	trẻ
	Số ngày sử dụng trung bình (HS-HP)	1.0	ngày
5. VẬN ĐỘNG HIẾN TẶNG	Số lần tư vấn nhóm	10	lần
	Số sự kiện (có số người tham dự lớn hơn 10, bao gồm cả truyền thông đại chúng, các hoạt động tạo nhu cầu tại bệnh viện và cộng đồng)	0	lần
	Số bà mẹ tiếp cận được để tạo trao đổi về việc tham gia hiến sữa cho NHSM bởi cán bộ NHSM, cán bộ y tế khác, hoặc tình nguyện viên (theo hình thức nhóm, cá nhân, sự kiện)	94	bà mẹ
	Cá nhân	67	bà mẹ
	Nhóm	27	bà mẹ
	Sự kiện	0	bà mẹ
	Số bà mẹ tiềm năng	21	bà mẹ

Báo cáo vật tư tiêu hao

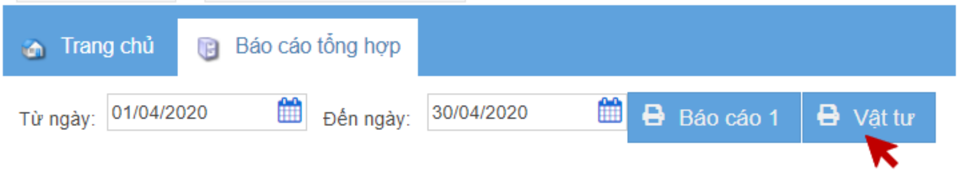

BÁO CÁO VẬT TƯ Từ ngày: 1/4/2020 đến ngày: 30/4/2020

Điểm	Phòng	OT 5mL	OT 10 mL	OT 20 mL		OT 50mL
				O	B	
SẢN G	Phòng 2	0	0	0	0	0
SẢN G	Phòng 1	0	0	0	0	0
KHU N	N 11	0	0	0	0	0
KHU N	N 10	0	0	0	0	0
KHU N	N 9	0	0	0	0	0
KHU N	N 8	0	0	0	0	0
KHU N	N 5	0	0	0	0	0
KHU N	N 7	0	0	0	0	0
SƠ SINH	PHÒNG 7	22	14	4	0	0
SƠ SINH	PHÒNG 14	152	136	122	0	92
SƠ SINH	PHÒNG 3	28	10	18	0	0
SƠ SINH	KANGAROO	209	266	364	0	239
SƠ SINH	PHÒNG 2	0	2	0	0	0
SƠ SINH	PHÒNG 15	54	62	66	0	54
SƠ SINH	PHÒNG 5	28	18	24	0	0
SƠ SINH	PHÒNG 9	52	58	94	0	74
SƠ SINH	PHÒNG 16	70	83	52	0	30
SƠ SINH	PHÒNG 11	32	51	56	0	18
SƠ SINH	PHÒNG 12	22	30	20	0	10
SƠ SINH	PHÒNG 10	46	24	8	0	0
KHU H	H 3	0	0	0	0	0
KHU H	H 6	0	0	0	0	0
KHU H	H 5	0	0	0	0	0
HẬU PHẪU	HẬU PHẪU	0	0	0	0	0
		**715**	**754**	**828**	**0**	**517**

Lưu file: Huỳnh Đức Duy
